# Near-infrared light activatable chemically induced CRISPR system

**DOI:** 10.1038/s41377-025-01917-8

**Published:** 2025-07-01

**Authors:** Lei Zhang, Xuejun Zhang, Le Qiu, Song Mao, Jia Sheng, Liming Chen, Umar Khan, Paul K. Upputuri, Yuri N. Zakharov, Mark F. Coughlan, Lev T. Perelman

**Affiliations:** 1 Center for Advanced Biomedical Imaging and Photonics, Division of Gastroenterology, Department of Medicine, Beth Israel Deaconess Medical Center, Harvard University, Boston, MA USA; 2 The RNA Institute, Department of Chemistry, University at Albany, Albany, NY USA

**Keywords:** Biophotonics, Optical manipulation and tweezers

## Abstract

The biggest challenge in using CRISPR technologies, which limits their widespread application in medicine, is off-target effects. These effects could, in principle, be minimized by ensuring that CRISPR is activated primarily in the targeted cells, thereby reducing the likelihood of unintended genetic modifications in non-target tissues. Therefore, the development of a light activatable CRISPR approach to dynamically control gene activation in both space and time would be highly beneficial. A drawback is that the overwhelming majority of recently introduced light activatable CRISPR systems require UV or blue light exposure, severely limiting the penetration depth of light in tissue at which CRISPR can be activated, and, in the case of UV light, raising safety concerns. A small number of systems that activate CRISPR using longer wavelengths are hindered by either slow light activation or issues related to toxicity and biocompatibility of the proposed techniques in humans. To address this, we developed a split-Cas9/dCas9 system in which activation is achieved through a near-infrared photocleavable dimerization complex. This photoactivation method can be safely used in humans in vivo, easily adapted to different split-Cas9/dCas9 systems, and enables rapid, spatially precise light activation across various cell types.

## Abstract (web summary)

Upon NIR illumination, photocleavable complexes release rapamycin, reactivating dCas9 via fragment reassociation to enable sgRNA-guided gene regulation with spatial control, allowing precise “writing” with NIR light.

## Graphical abstract

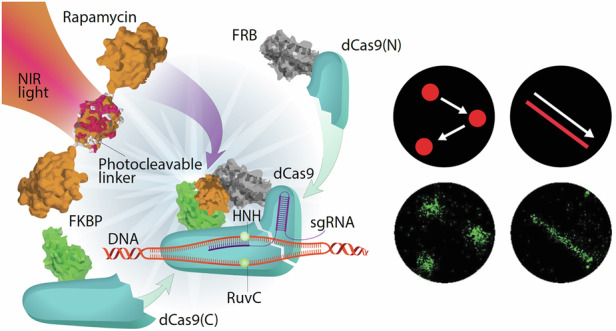

## Introduction

The development of CRISPR (clustered regularly interspaced short palindromic repeats) technology has provided a promising approach for controlling gene expression^1–4^. However, one of the biggest challenges in applying CRISPR technologies to gene therapy, precision medicine, and disease research is the risk of off-target effects. Both genetic and epigenetic CRISPR-based modifications are currently considered too risky for use on a whole-organism scale, necessitating the localization of their activity. This can be achieved by ensuring that CRISPR is activated only in the cells that require modification, reducing the likelihood of unintended genetic alterations in non-target cells^5^.

Introduced in the last decade, photoactivatable CRISPR offers the potential to perform localized genetic or epigenetic alterations without the risk of organism-scale off-target modification. Initial photoactivatable CRISPR systems utilized the light-inducible heterodimerizing calcium and integrin-binding protein 1 (CIB1) and cryptochrome 2 (CRY2) proteins. CRY2 was linked to a transcriptional activation domain such as VP64 or p65 and CIB1 was fused to Cas9 or dCas9 (an inactive, dead Cas9) either to one terminal (Cas9/dCas9-CIB1)^6,7^ or to both the N- and C-termini (CIBN-Cas9/dCas9-CIBN)^8^. When exposed to blue light, CRY2 and CIB1 heterodimerize and, guided by the single guide RNA (sgRNA), bind to the target DNA sequence to regulate gene expression. Later, a photoactivatable system using split dCas9 (N-terminal and C-terminal halves) was developed^9,10^. By fusing Magnets^11^, a photoactivatable dimerization domain, to each fragment of the Cas9 or dCas9, researchers showed genome editing or optogenetic control of RNA-guided transcription interference. Unlike the above strategies that used photoactive proteins, a different approach using chemistry to photocage CRISPR was also established. To enable conditional regulation of the CRISPR system, a light-responsive Cas9 was engineered by introducing a caged lysine amino acid at a specific site. When exposed to UV light, photocaged Cas9 exhibited cleavage activity comparable to that of wild-type Cas9^12^.

Unfortunately, all of the light-activated CRISPR approaches mentioned above require UV exposure to bind split Cas9 or dCas9 components. Not only could the UV irradiation have phototoxic effects on cells and tissue^13^, but, due to higher scattering in the UV spectral region, it also severely limits the depth in tissue at which CRISPR can be photoactivated^14^. Last but not least, all the mentioned photoactivatable mechanisms require engineering plasmid-coded photoactivation, which presents considerable challenges, such as limited efficiency of transformation or low reproducibility of results. To address the latter problem, an alternative approach to photoactivation called CRISPR-plus was introduced^15^. This approach uses photocleavable oligonucleotides that hybridize with target regions of the sgRNA, blocking its interaction with the target DNA. Photolysis then releases the oligonucleotides, enabling the sgRNA to bind to the DNA. The photoactivatable protector sgRNA complex can be synthesized outside of the cell and does not require the plasmid for delivery, making CRISPR photoactivation easier. Unfortunately, the CRISPR-plus method does not provide a fully discrete on/off mechanism and the synthesized photocleavable nucleotides still require irradiation with UV light. Almost all recent attempts to accelerate activation to under one minute^16^ have required the use of short-wavelength UV and blue light, which significantly reduce the potential penetration depth of activation and raise safety concerns.

Recently, activation using longer wavelengths of 660 nm^17^ and 730 nm^18,19^ has been demonstrated. However, both techniques were limited by slow light activation, with the former requiring 1 hour of irradiation and the latter taking up to 6 hours for activation. Activation using an even longer wavelength of 980 nm^20,21^ with a relatively short 20-minute irradiation, on the other hand, required the use of upconversion nanoparticles. While these nanoparticles show promise, no upconversion nanoparticle-based products have been approved for human use to date due to concerns about toxicity and biocompatibility. Another type of nanoparticle, cationic polymer-coated gold nanorods, has been recently employed to activate CRISPR within 5 minutes of exposure to 1064 nm light, by increasing the local temperature to 42 °C and employing a heat-inducible promoter, HSP70^22^. However, keeping the local temperature of cells within the very narrow HSP70 optimal activation temperature range of 41.5 to 42.0 °C is a rather difficult task and the approach is limited by low transfection efficiency^23^. Finally, a phytochrome-based photoswitch (REDMAP) system was reported to be able to induce transcriptional activation within seconds of illumination^24^, however, it requires simultaneous irradiation with two visible wavelengths of 660 nm and 730 nm and the external supply of exogenous chromophores limiting its therapeutic prospects^24^.

A potential reason why the development of a rapid near-infrared (NIR) activatable CRISPR appears to be elusive is the need to combine several somewhat conflicting properties in a single molecular complex. For example, NIR activation would most likely require employing heptamethine cyanine photochemistry^25^, however, combining a heptamethine cyanine-based photocleavable complex with a split-dCas9/Cas9 system is nontrivial, especially if there is a requirement for rapid uncaging. Therefore, we decided to compartmentalize the problem, developing a two-stage system which combines light activation with chemically induced dimerization. An attractive option for a chemical dimerizer, which is not only biocompatible but can be used in vivo and is even capable of crossing the blood–brain barrier, is rapamycin^26–28^. Rapamycin can bind the FK506 binding protein (FKBP) through the FKBP rapamycin binding (FRB) domain^29^ which in turn could be fused to two split dCas9 fragments called C- and N-terminal fragments^30^. On the other hand, a UV light-cleavable rapamycin dimer has already been developed^31^, paving the way for replacing the existing UV light-cleavable cage with the heptamethine cyanine-based photocleavable cage. The resulting system could be very adaptable as photoactivation and dimerization are separated.

**Fig. 1:**
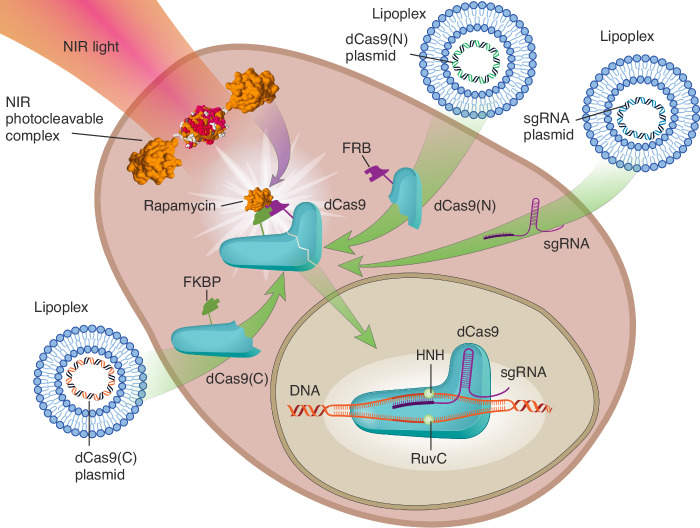
Schematic of the NIR light activatable chemically induced CRISPR system. Lipoplex vectors expressing dCas9 fragments and sgRNA are cotransduced into the cell, while photocleavable complexes also cross the cell membrane and are incorporated in the cytoplasm. Upon NIR illumination, photocleavable complexes release rapamycin molecules which reassociate C- and N- terminal fragments of dCas9 fused to the FKBP and FRB domains, reconstituting the ability of transcription activation/repression guided by sgRNA

The schematic of the two-stage light activation with chemically induced dimerization system is presented in Fig. 1. Here, upon NIR illumination, photocleavable complexes rapidly release rapamycin molecules. The released rapamycin monomers reassociate C- and N- terminal fragments of dCas9 fused to the FKBP and FRB domains, reconstituting the ability of transcription activation/repression guided by sgRNA. Due to the ability of NIR light to propagate deep into tissue, this approach can allow spatially localized CRISPR activation far from the activation light source. Furthermore, by scanning tissue with the illumination beam in a precise pattern, it should be possible to achieve highly localized CRISPR-mediated gene regulation without the need for a photomask, making it particularly promising for translational applications. This could be vital to the development of targeted photo-epigenetic therapies without side effects in healthy tissue.

## Results

### Heptamethine cyanine based photocleavable rapamycin dimer complex

**Fig. 2:**
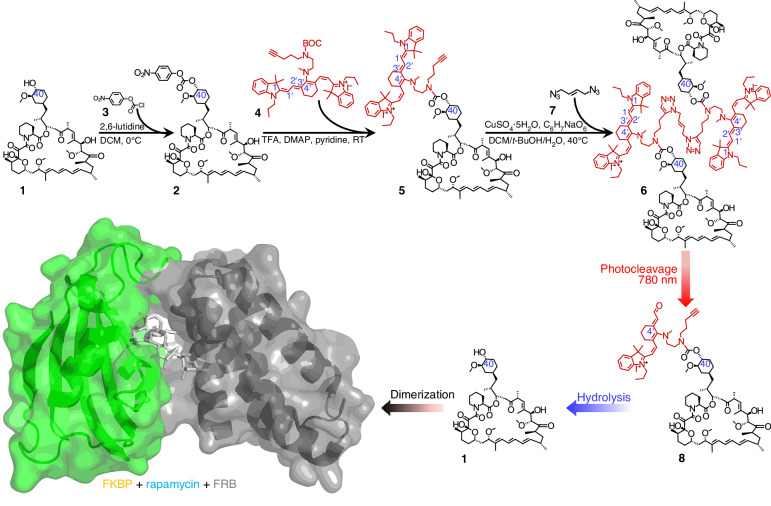
Synthesis and photoactivation of IR780-bridged rapamycin. IR780 is shown in red. FKBP12 (green) and FRB (gray) form a heterodimer upon light-induced photocleavage of rapamycin (shown in light blue) from the dimer. The FKBP-rapamycin-FRB complex structure (PDB ID: 3FAP) was from the Protein Data Bank

In order to synthesize a new NIR photoactivatable CRISPR system which can be safely used in humans in vivo, we employed an uncaging strategy^25^ that relies on cyanine photobleaching via a regioselective photooxidative polyene cleavage reaction and utilizes the commercially available NIR lipophilic cation heptamethine fluorescent reagent IR780 (Sigma-Aldrich). Although photobleaching is typically considered a drawback in many other applications, it is the central component of our uncaging strategy. We modified rapamycin **1** (Sigma-Aldrich), a biocompatible compound approved for clinical use in humans, by conjugating it with IR780 (Fig. 2). This modification prevents rapamycin from inducing Cas9 split-dimerization in the NIR-activatable CRISPR system until it is exposed to NIR light. Earlier research has shown that the C-40 hydroxyl group of rapamycin is essential for forming a hydrogen bond with glutamine 53 of FKBP12^32,33^. Based on this, we hypothesized that attaching IR780 at the C-40 position of rapamycin **1** would disrupt this hydrogen bond, thereby preventing the FKBP-FRB protein interaction. To test this, we synthesized 4-nitrophenyl (PNP)-activated rapamycin **2** (see Supplementary Note 1 and Scheme S1 for synthesis procedure) by selectively acylating the C-40 hydroxyl group with 4-nitrophenyl chloroformate **3** and 2,6-lutidine^34^. The identity of PNP-activated rapamycin **2** was confirmed by 1H NMR and HRMS analysis (Supplementary Figs. S1 and S2). Furthermore, prior studies have shown that NIR light causes cleavage of the polymethine chain in IR780 at the C1−C1′ and C2′−C3′ bonds, leading to subsequent hydrolysis at the C4′−N position^25,31,35^. To enable the conjugation of IR780 with PNP-activated rapamycin and facilitate the NIR light-triggered release of rapamycin via hydrolysis, we synthesized a carbamate linkage^35^. This process began with commercially available 4-pentyn-1-ol, which was tosylated, then the tosyl group was replaced by ethanolamine. After Boc-protection of the nitrogen (Supplementary Fig. S3), the hydroxyl group (-OH) was converted into an amino group (-NH2) (see Supplementary Note 1 for synthesis procedure and Supplementary Fig. S4). The resulting carbamate (Supplementary Fig. S5) was then conjugated with IR780 to form a C4′−N linked IR780-carbamate compound **4** (Supplementary Figs. S6 and S7). After removal of the Boc group using trifluoroacetic acid (TFA), the deprotected amine reacted with the carboxyl group of PNP-activated rapamycin **5** in the presence of DMAP and pyridine (Supplementary Figs. S8 and S9). To completely prevent dimerization of the monomer until exposure to NIR light, we synthesized IR780-bridged dimers **6** to significantly enlarge the caging group^31^ using an efficient click reaction (Fig. 2 and Supplementary Figs. S10**7**, S11 and S12). The compound is soluble in DMSO and pure ethanol and is highly stable, maintaining its properties after being stored in aqueous solution at -20 °C for over a year. When exposed to NIR light, the dimer undergoes photocleavage, releasing rapamycin along with a residue **8**. This residue is then removed through hydrolysis, allowing rapamycin to bind to FKBP and FRB. Together, these processes enable precise, light-controlled activation of the dimer for biological applications.

**Fig. 3:**
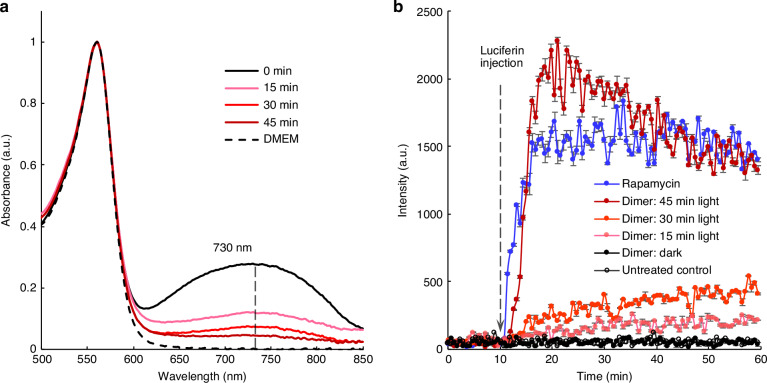
Characterization of the light-cleavable rapamycin dimer complex. **a** Absorbance spectra of 4 µM light-cleavable dimer dissolved in DMEM basal medium, illuminated for 15, 30, and 45 minutes, respectively. The spectra are normalized to the 560 nm peak. **b** Luminescence signals of HEK293FT cells co-transfected with plasmids encoding FKBP-SmBit and FRB-LgBit (Promega) and treated with 30 nM rapamycin (positive control), 15 nM dimer without illumination, and 15 nM dimer illuminated for 15, 30, and 45 minutes, respectively. Negative control: untreated cells. Error bar: standard deviation calculated from three independent samples

To investigate the optical characteristics of the heptamethine cyanine-based photocleavable rapamycin dimer complex and its cleavage efficacy, we measured the absorption spectrum of the dimer after illuminating it with a custom-built LED array (see Materials and Methods, Array-based Photoactivation Setup and Supplementary Fig. S13). The dimer (4 µM) was dissolved in DMEM medium containing phenol red, and the absorption spectra were collected using a microvolume spectrophotometer (Thermo Fisher Scientific) at different time points following illumination (0, 15, 30, and 45 min). The spectra showed a heptamethine cyanine absorption band from 620 to 820 nm, along with the phenol red absorption bands at 450 and 560 nm (see Fig. 3a). After 15 minutes of illumination, the heptamethine cyanine absorption band decreased significantly, indicating that more than half of the photocleavable rapamycin dimer was photobleached and cleaved after approximately 15 minutes of exposure (Fig. 3a).

To investigate whether the dimer could prevent FKBP-FRB dimerization yet still be cleaved into monomeric rapamycin to induce dimerization upon NIR illumination in living cells, we performed a NanoBiT complementation-based protein-protein interaction assay using human embryonic kidney (HEK) 293FT cells. This assay relies on the recombination of two complementary subunits of the NanoLuc luciferase (FKBP-SmBit and FRB-LgBit, Promega). In the presence of rapamycin, the subunits can dimerize, forming active luciferase and generating luminescent signals^36^. It demonstrated that the dimer bridged with IR780 could inhibit FKBP-FRB dimerization (Fig. 3b). This is supported by the induction of luminescence with the addition of rapamycin (30 nM) and the observation that the equivalent concentration of rapamycin dimer did not lead to any detectable luciferase expression, thus showing no background luminescence in the absence of photoinduction (Fig. 3b). In subsequent experiments, we tested whether NIR-photolyzed rapamycin dimer could release native rapamycin capable of inducing FKBP and FRB dimerization in living cells by treating HEK293FT cells expressing FKBP-SmBit and FRB-LgBit with NIR-photolyzed rapamycin dimer. This revealed that in living cells, split luciferase proteins could be fused by the released rapamycin, producing luminescence after substrate (luciferin) administration (Fig. 3b). Cells treated with the dimer and subjected to 45 minutes of illumination generated luminescence similar to cells treated with pure rapamycin. These results demonstrate the feasibility of using the IR780-bridged rapamycin dimer for optogenetic control of CRISPR-dCas9 activity with NIR light.

### NIR photoactivated CRISPR-mediated gene expression in live cells

**Fig. 4:**
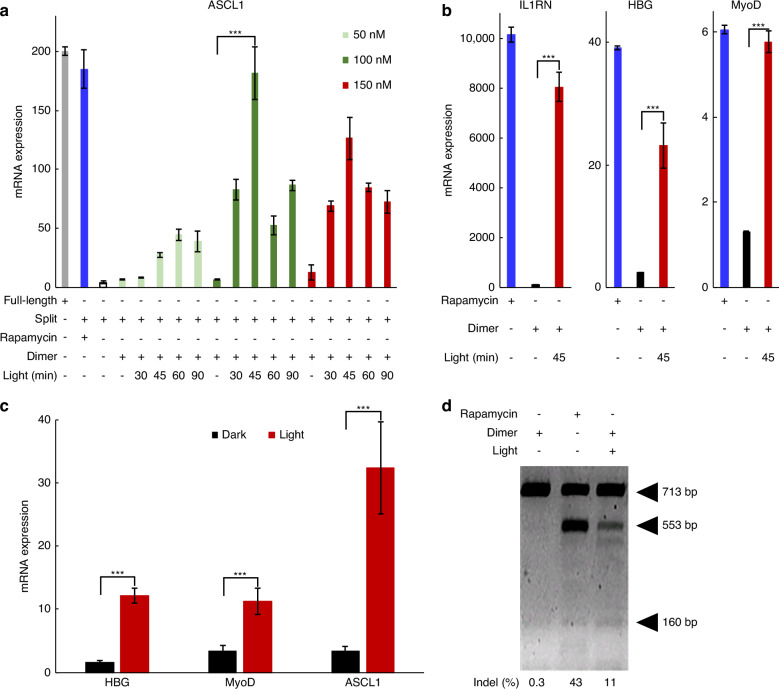
Light-inducible modulation of endogenous human genes. **a** *ASCL1* activation in HEK293FT cells co-transfected with split dCas9 and sgRNAs followed by treatment with different concentrations of dimer for different times of near-infrared light illumination. **b** *IL1RN*, *HBG* and *MyoD* expression in HEK293FT cells after near-infrared light treatment. **c** Multiple target genes were activated using sets of three sgRNAs designed to target *HBG*, *MyoD* and *ASCL1* in HEK293FT cells. **d** Light-activated CRISPR-based editing of the *OPTN* gene in HeLa cells. Arrowheads indicate the putative light-activated CRISPR cleavage site. Cells without illumination served as a negative control and full-length dCas9 served as a positive control. *** *P* < 0.001

To test whether the dimer can induce gene expression through CRISPR-mediated activation using NIR light, we examined the expression of an endogenous gene by transfecting HEK293FT cells with split dCas9 and a set of four sgRNAs targeting the human *ASCL1* promoter^30^. Following transfection, the cells were subjected to NIR illumination. To optimize the dimer concentration and illumination timing for efficient *ASCL1* activation, illumination treatments began 24 hours post-transfection. The cells were exposed to various dimer concentrations and illuminated with NIR light for varying durations, up to 90 minutes, allowing for a systematic evaluation of activation efficiency. After illumination, the cells were incubated in the dark for an additional 24 hours, qRT-PCR was then conducted to measure *ASCL1* expression. Cells exposed to illumination exhibited significantly higher mRNA levels compared to those maintained in darkness (*P* < 0.001). Furthermore, we observed both light-dependent (*P* < 0.001) and concentration-dependent (*P* < 0.001) activation (Fig. 4a). Cells treated with 100 nM rapamycin dimer and illuminated for 45 minutes exhibited activation levels similar to those induced by full-length dCas9 or rapamycin (*P* = 0.434 and 0.881, respectively). Importantly, in transfected cells treated with the dimer but not exposed to illumination, mRNA remained at levels comparable to those observed in mock-transfected cells, with no significant differences (*P* = 0.605, 0.668, and 0.079, respectively). This result demonstrates minimal background activity in the “off” state when photoinduction is absent.

To demonstrate the system’s potential, we tested multiple endogenous gene targets by delivering the NIR-mediated CRISPR-dCas9 system with groups of sgRNAs targeting human *IL1RN*, *HBG* and *MyoD*^37^ in HEK293FT cells (Fig. 4b). Illuminated cells showed a significant increase in mRNA levels compared to both untransfected cells and transfected cells kept in the dark for all tested genes (one-way ANOVA: *IL1RN P* < 0.001, *HBG P* < 0.001, *MyoD P* < 0.001). The activation levels were comparable to those achieved using full-length dCas9 or rapamycin. Background activity remained minimal, as reflected in the high +NIR/–NIR activation ratios: *IL1RN* = 68.5, *HBG* = 9.3, and *MyoD* = 4.5. We also evaluated the capability of the NIR-mediated CRISPR-dCas9 system to simultaneously regulate the expression of multiple genes. By delivering sets of three sgRNAs targeting *ASCL1*, *HBG*, and *MyoD*, we successfully activated all target genes (Fig. 4c). Notably, the activation levels of each gene varied between the multiplexed and single-gene activation experiments, suggesting potential variability in target efficiency when multiple genes are addressed simultaneously. These results highlight the potential of the NIR-mediated CRISPR-dCas9 system for multiplexed activation of endogenous genes through the concurrent delivery of multiple sgRNAs.

To demonstrate the feasibility of using the photoactivatable approach for light-dependent genome editing, we transfected HeLa cells with split Cas9 and a sgRNA targeting the human *OPTN* locus. Deletion mutations induced by light exposure were detected using the SURVEYOR nuclease assay (Fig. 4d). In the absence of light, cells transfected with split Cas9 targeting *OPTN* exhibited only a 0.3% indel rate. However, after light irradiation, these cells showed significantly higher indel rates at the *OPTN* locus. Sanger sequencing further confirmed that NIR-induced indel mutations occurred specifically at the targeted *OPTN* locus, validating the precision of this light-controlled genome editing system.

### Rapid and spatially precise activation of CRISPR with NIR light

**Fig. 5:**
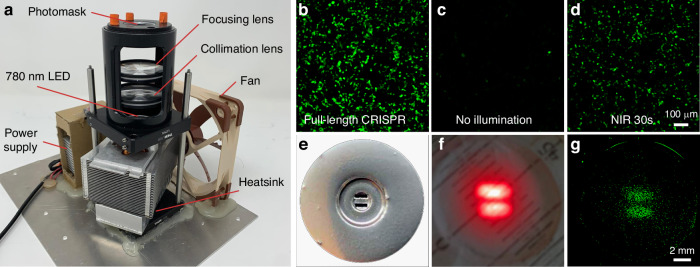
Localized CRISPR photoactivation. **a** Custom LED illumination setup. **b** Fluorescence of eGFP-expressing HEK293FT cells treated with rapamycin dimer, transfected with full-length dCas9. At the top of the setup there is a holder for a 35 mm glass-bottom dish. The 3D-printed double slit screen is located in the focal plane of the illumination setup. **c** Fluorescence of eGFP-expressing HEK293FT cells treated with rapamycin dimer, transfected with split dCas9 without NIR illumination. **d** Fluorescence of eGFP-expressing HEK293FT cells treated with rapamycin dimer, transfected with split dCas9 under 30 seconds of NIR illumination. **e** 3D-printed double-slit screen with each slit being 500 μm wide and 2 mm long. **f** NIR illumination using double slit screen. **g** Corresponding pattern of the fluorescence of eGFP-expressing cells

We tested the capabilities of a heptamethine cyanine-based photocleavable system to achieve rapid and spatially precise activation of CRISPR-dCas9 in living cells using NIR light. To visualize CRISPR activation with NIR light we employed a single-LED illumination setup with a photomask (see Materials and Methods, Photomask-based Photoactivation Setup) delivering light onto cells located in a 35-mm glass-bottom dish (Mattek). We employed an eGFP reporter system that produces eGFP in the presence of dCas9^6^. HEK293FT cells were co-transfected with dCas9 split constructs, the eGFP reporter plasmid, and a sgRNA construct targeting the eGFP reporter. Prior to illumination, the cells were treated with 100 nM rapamycin dimer. We developed a digitonin-based reversible permeabilization method (Supplementary Note 2) to facilitate the passage of the IR780-bridged rapamycin dimer through the cell membrane, which further reduced the required illumination time. When incubated in the dark, transfected cells treated with the rapamycin dimer exhibited eGFP levels similar to those of control cells transfected with an empty plasmid. However, cells illuminated with NIR light for 30 seconds using the custom LED illumination setup shown in Fig. 5a demonstrated eGFP fluorescence comparable to that achieved with full-length CRISPR activation (Fig. 5b–d). To demonstrate the ability of the NIR light activatable CRISPR-dCas9 system to achieve spatially precise light activation, we then illuminated HEK293FT cells co-transfected with dCas9 split constructs, the eGFP reporter and its sgRNA through a 3D-printed double-slit screen, where each slit was 500 μm wide and 2 mm long (Fig. 5e, f). The resulting pattern of eGFP fluorescence in the cells closely matched the double-slit screen pattern (Fig. 5g).

**Fig. 6:**
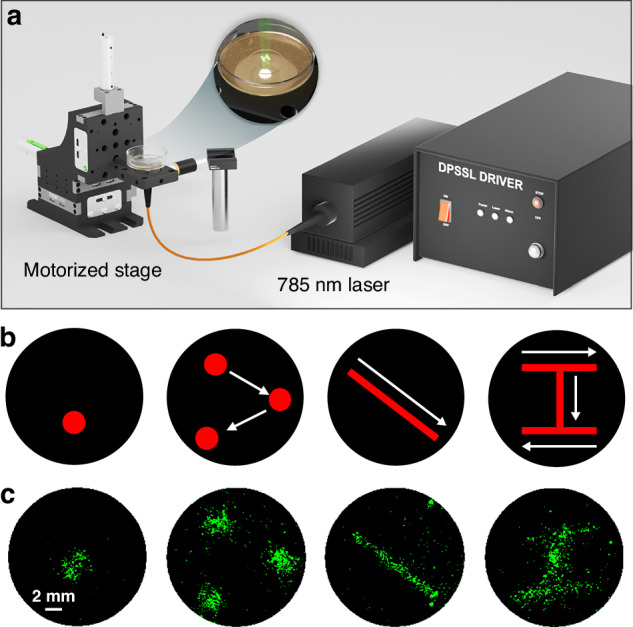
Writing with NIR light activatable CRISPR using laser scanning. **a** Optical fiber-coupled NIR illumination setup with fiber-coupled laser, a multimode fiber, a collimator, a convex lens and a motorized XYZ stage. **b** Several pre-programmed illumination patterns. **c** The same patterns visualized with eGFP fluorescence. Scale bar: 2 mm

Most methods of CRISPR photoactivation employ photomasking^6,9,10,18,19,24^. However, photomasking approaches lack the ability to rapidly adjust the illumination patterns. To enable real-time control over illumination patterns, we replaced photomasking with laser scanning (see Materials and Methods, Laser Writing Photoactivation Setup and Fig. 6a). The 785 nm continuous-wave (CW) mode-locked laser (MDL-III-785-450mW, CNI) illuminated the sample of HEK293FT cells transfected with split dCas9 constructs, the eGFP reporter plasmid, and the sgRNA construct targeting the reporter. After 24 hours transfection, samples were illuminated with the programmable motorized stage, which produced various illumination patterns (Fig. 6b) which triggered eGFP expression (Fig. 6c). The entire “writing” process with NIR light activatable CRISPR takes less than 10 seconds and does not require any increase in light intensity.

## Discussion

We developed an NIR photoactivatable CRISPR-Cas9/dCas9 system that enables rapid, spatially precise, and efficient gene editing and epigenetic modulation. This system allows for light activation across various cell types and can be safely used in humans in vivo. It achieves high levels of transcriptional activation, which is comparable in some cases to full-length dCas9. The system is simpler than light-activated CRISPR systems that rely on the association of paired photoswitchable proteins. Additionally, it uses safe NIR light, unlike some proposed photoactivatable CRISPR systems that employ UV radiation, and it does not rely on compounds that may raise concerns about toxicity and biocompatibility. Due to its use of the NIR wavelength, this photoactivatable CRISPR system can provide deep tissue activation.

It is important to note that the developed chemically inducible dimerization system allows for faster activation compared to many existing approaches, with spatiotemporal regulation achieved within just 10 seconds of illumination, triggering an “unclick” reaction. Our approach uses a bifunctional small molecule to rapidly and specifically assemble proteins^38^, offering quick onset and precise control. While chemically inducible dimerization has been used in some previously described systems—such as doxycycline-modulated CRISPR/Cas9 expression^39^, rapamycin-inducible split-Cas9^30^, and transient delivery of Cas9:sgRNA complexes^40^—these approaches are limited by concerns over chemical diffusion, which hinders spatiotemporal genome editing. To address this limitation, we integrated NIR light-activated molecules (IR780) with rapamycin to block the binding domains for FKBP and FRB, which are fused to split dCas9 or Cas9. By inducing IR780 photolysis with precise NIR light illumination, rapamycin monomers were released in a spatially and temporally controlled manner. This enabled localized dCas9 activation exclusively in illuminated regions (as shown in Fig. 6), with no detectable effects in unilluminated areas or control groups. The rapid and localized nature of the illumination mitigates concerns about chemical diffusion^9^, while the precise control of cell temperature ensures no damage during CRISPR activation.

Digitonin is a cholesterol-solubilizing agent that can preferentially permeabilize cholesterol-rich plasma membrane while leaving other membranes intact^41^. Studies have shown that nontoxic concentrations of digitonin can significantly enhance drug efficacy, making it a valuable tool for increasing drug absorption into the cytoplasm, particularly in cancer treatments^42^. To facilitate the entry of the IR780-bridged rapamycin dimer into cells, we developed a safe and reversible permeabilization method using digitonin to enlarge membrane pores. GFP expression-based cell viability tests confirmed that this method caused no detectable damage or defects in the tested cells, demonstrating its safety and reliability.

Existing light-activated CRISPR systems that use UV light can achieve activation within several minutes^12,15,43^, while blue light-based systems typically require much longer activation times, ranging from several hours to tens of hours^6–10^. However, both UV and blue light have very limited tissue penetration. In contrast, red and especially NIR light can penetrate tissue significantly deeper. While activation times for red and NIR systems have typically been in the range of many hours, more recent work has demonstrated activation within minutes^17–24,44^. Some early systems were designed specifically for use in Cre-dependent cellular or genetic contexts^23^, but more recent platforms support broader gene-targeting capabilities and are compatible with a wide range of cell types. These newer systems are also often associated with minimal or negligible cytotoxic effects. Nonetheless, the phototoxicity of UV light continues to limit the in vivo applicability of UV-based systems, while concerns about nanoparticle biocompatibility affect the clinical translation of nanoparticle-based approaches. In contrast, our approach enables rapid activation in just 10 seconds using an NIR-photocleavable dimerization complex based on rapamycin that is not only biocompatible and suitable for in vivo use but also capable of crossing the blood–brain barrier.

Taking into account the attenuation of NIR light in tissue^45^ due to scattering and absorption, the CRISPR-Cas9/dCas9 system described here could be photoactivated at depths of several millimeters using realistic illumination powers of 0.5 to 1 mW mm^-^² at the tissue surface. Even deeper photoactivation, or activation at lower power thresholds, may be achievable through two-photon absorption in the short-wavelength infrared (SWIR) region, specifically at 1.55 μm, where light scattering is significantly reduced. Two-photon absorption and emission of heptamethine cyanine dyes at this wavelength have been the subject of recent investigations^46,47^, supporting their potential for future photocleavage applications.

Unlike other photoswitchable CRISPR systems that rely on photomasking for localized CRISPR activation, we developed a scanning laser beam approach, which allows “writing” with NIR light activatable CRISPR in a pre-programmed illumination patterns without the need for a photomasks. Photomasks are inflexible and require predesign, limiting their ability to quickly adjust spatial patterns. In contrast, our illumination system can both be easily automated or operated manually by a user changing CRISPR activation patterns on the fly, offering greater flexibility and potential for translational applications.

In conclusion, the NIR photoactivatable CRISPR-Cas9/dCas9 system we developed offers a robust method for activating CRISPR-Cas9/dCas9 functions with precise spatial and temporal control within seconds, without requiring complex plasmid engineering. Furthermore, it facilitates multiplex gene regulation through the combination of sgRNAs. This approach holds significant potential for applications in basic and translational sciences where precise spatiotemporal control of gene modulation in cells or tissues is essential, while also minimizing off-target effects associated with CRISPR-Cas9 or dCas9.

## Materials and methods

### Cell culture

HEK293FT cells (Thermo Fisher Scientific) and HeLa cells (ATCC) were cultured as previously described^48,49^.

### Luminescence assay

Cells were analyzed using the NanoBiT® protein-protein interaction system (Promega) following a protocol adapted from the manufacturer’s instructions. HEK293FT cells were seeded on day 0 at 3 × 10^5^ cells mL^-1^ density in 96-well plates (Corning). After 24 hours (day 1), we transfected cells with vectors encoding FKBP-SmBiT and FRB-LgBiT. On day 2, the dimer photolyzed for varying durations was added, and the expression of NanoLuc luciferase or NanoBiT® fusion proteins was measured using the NanoGlo® luciferase assay kit (Promega) according to the manufacturer’s protocol. Rapamycin (30 nM) was used as a positive control. SpectraMax iD3 multi-mode microplate reader (Molecular Devices) was used to measure luminescence after equilibrating the plates at 37 °C for 10–15 minutes.

### Plasmids

The plasmids encoding Cas9 (PX851, #62883 and PX852, #62884), split dCas9 (PX855, #62887 and PX856, #62888), dCas9-VP64 (#61425), an eGFP-expressing surrogate reporter (#60718), and a sgRNA targeting the eGFP reporter (#60719) were obtained from Addgene. The sgRNA expression construct was prepared following a previously described protocol^50^. sgRNAs targeting *ASCL1*, *IL1RN*, *HBG*, and *MyoD* were generated by cloning annealed oligonucleotides (IDT) into the BbsI site of the Addgene MS2 plasmid (#61424). Successful constructions were verified by Sanger sequencing (Genewiz) using U6 primers. Guide sequences used in the current study are provided in Supplementary Table S1.

### Optogenetic gene activation

HEK293FT cells were seeded at 2.5 × 10^5^ cells per well in a 24-well plate on day 0. After 24 hours (day 1), cells were transfected with 600 ng of plasmids per well containing sgRNA and split dCas9 at a mass ratio of 1:2:2. For positive controls, dCas9-VP64 expression plasmid was transfected at a mass ratio of 2:1 relative to each sgRNA expression plasmid, while 600 ng of pUC19 empty plasmid was transfected as a negative control. For eGFP activation experiments, the eGFP-expressing surrogate reporter plasmid (100 ng), sgRNA plasmid (300 ng), and split dCas9 plasmids (200 ng each of PX855 and PX856) were co-transfected. Positive controls included 200 ng of dCas9-VP64 expression plasmid transfected with 100 ng of eGFP reporter plasmid, 300 ng of sgRNA plasmid, and 200 ng of pUC19. Transfections were performed using TransIT-X2 (Mirus).

After 24 hours of transfection (day 2), split-Cas9 dimerization was induced by either a rapamycin dimer illuminated with NIR light or 200 nM rapamycin (Sigma). Cells were harvested on day 3 for gene expression analysis via qPCR or fixed with 4% paraformaldehyde (PFA) for imaging.

### Photoactivation setups

Several photoactivation setups, each tailored to a specific purpose, were constructed to study the rapid and biocompatible NIR activation of CRISPR developed here.

Array-based Photoactivation Setup. This LED array-based setup (Supplementary Fig. S13) delivered approximately 30 mW cm^−2^ of power to the sample and employed six 730 nm LEDs (LST1-01G01-FRD1-00, Opulent Americas) pre-mounted on a 130 mm × 90 mm aluminum board. The LEDs were wired in two series, with each series connected in parallel on the same aluminum board. The array was powered by a DC Power Supply (TP3016M, Tekpower). During illumination, a 24-well plate was positioned 1 cm above the LED array, allowing upward-directed light to uniformly illuminate the bottom of the middle 12 wells of the plate.

Photomask-based Photoactivation Setup. This high-power, single LED setup (Fig. 5a) was developed to use a longer wavelength, shorten illumination times, and incorporate photomasks. It delivered approximately 80 mW cm^−^² of power to the sample and employed one 780 nm LED (LED Engin) mounted on an aluminum heatsink. To mitigate heat generation, a fan was installed on the side of the heatsink. The illumination spot size of approximately 6.25 mm² and power density were adjusted using collimation and focusing Fresnel lenses (FRP232, Thorlabs). A photomask, designed in SolidWorks and fabricated using a 3D printer (Form 2, Formlabs), was installed at the top of the setup, below the holder for a 35 mm glass-bottom dish for cell cultures.

Laser Writing Photoactivation Setup. The laser writing photoactivation setup (Fig. 6a) utilized a 785 nm CW mode-locked laser (MDL-III-785-450mW, CNI) and a motorized XYZ stage (MT3-Z8, Thorlabs). The multimode optical fiber (365 µm core, 0.39 NA) transmitted laser light to a fiber collimator (F240SMA-780, Thorlabs) and an aspheric lens (A240TM-B, Thorlabs), both mounted on a motorized stage. The size of the illumination spot on the sample was adjusted by the vertical movement of the stage. Various CRISPR activation patterns were produced by horizontal stage movement. Both stage movements in the XYZ directions and laser power were controlled using a custom-built graphical user interface (GUI) with MATLAB.

### Gene expression analysis by qPCR

We used Trizol (Life Technologies) to isolate total RNA from harvested cells. A NanoDrop™ One^C^ microvolume UV-Vis spectrophotometer (Thermo Fisher Scientific) was used to assess the quality and quantity of the isolated RNA. One microgram of RNA was utilized for reverse transcription using a high-capacity cDNA reverse transcription kit (Thermo Fisher Scientific). QPCR analysis was performed in a reaction of 10 µL with TaqMan qPCR probes and fast advanced Master mix (Life Technologies) on a QuantStudio 6 flex PCR system (Applied Biosystems) according to the manufacturer’s specifications. Data was analyzed by the ΔΔC_t_ method^51^ and GAPDH was used as the internal control gene.

### SURVEYOR assay to detect genomic modifications

Surveyor assays were performed using the GeneArt Genomic Cleavage Detection Kit (Thermo Fisher Scientific). Genomic DNA was extracted, and the region surrounding the CRISPR target site for *OPTN* was amplified by PCR using specific primers. The resulting PCR products were purified with the Monarch PCR & DNA Cleanup Kit (NEB). For T7 endonuclease I digestion, purified DNA (100 ng) was treated and examined on an agarose gel (2%). Ethidium bromide was used to stain the gels, and gel imaging was performed using a Gel Doc Imaging System (Bio-Rad). The indel rate was quantified by measuring the relative band intensities of the cleaved fragments compared to the total intensity of digested and undigested PCR products using ImageJ.

### Spatial photoactivation of GFP

HEK293FT cells were seeded onto 35-mm dishes containing a 13-mm No.0 coverslip precoated with poly-D-lysine (Mattek). After 24 hours, a total of 2000 ng of DNA was used for split-dCas9 transfection: 700 ng of eGFP reporter construct, 300 ng of sgRNA plasmid, 500 ng of dCas9(N)-FRB (PX855) and 500 ng of dCas9(C)-FKBP (PX856). After 24 h of transfection, cells were incubated in a hypotonic solution (Rainbow Scientific) containing digitonin at 37 °C for 15 minutes, followed by equilibration in DMEM containing the rapamycin dimer at 37 °C for 20 minutes. Following equilibration, the medium was replaced with fresh DMEM, and the cells were exposed to patterned illumination. Twenty four hours later, we fixed the cells using 4% PFA for imaging. As a positive control, transfected cells were incubated in a hypotonic solution (Rainbow Scientific) containing digitonin at 37 °C for 15 minutes. The medium was then replaced with DMEM containing rapamycin for 20 minutes of equilibration at 37 °C, followed by replacement with fresh DMEM. After an additional 24 hours, the cells were fixed with 4% PFA for imaging.

### Imaging and image stitching

The microscope (LSM, Carl Zeiss) was used to capture photomicrographs of green fluorescent protein (GFP) signals using a 5x objective. Each image (3200 × 2200 pixels, 2.806 × 1.929 mm scale) covered a single field of view. For larger samples exceeding the microscope’s field of view, images were taken at multiple positions and merged to create a high-field view of GFP expression. To automate this process, we developed a MATLAB program that identifies the sample’s top-left corner and initiates scanning. The image acquisition is fully automated from this starting point. A total of 8 × 8 images were scanned sequentially from left to right. To ensure proper coverage, the microscope moved 2.2 mm in the X direction or 1.5 mm in the Y direction between adjacent fields of view, maintaining approximately 20% overlap. Image mosaicking was performed using the open-source MIST^52^ plugin, included in the Fiji distribution of ImageJ. This tool blends the edges of overlapping sub-images to create a seamless composite, leveraging the intentional spatial overlap for accurate alignment.

### Statistics

SPSS (IBM) was used for statistical analyses, and data are shown as mean ± standard deviation (SD). Comparisons between two groups were made using an unpaired two-tailed Student’s t-test. For comparisons involving more than two groups or factors, statistical significance was determined using one-way or two-way analysis of variance (ANOVA) followed by Tukey’s post hoc test. A *P*-value < 0.05 was considered statistically significant. Significance levels were indicated by asterisks: ***P < 0.001.

## Supplementary information

Supplementary Information for Near-infrared light activatable chemically induced CRISPR system

## Data Availability

The data supporting this study’s findings are available from the corresponding author upon reasonable request.

## References

[1] Jinek, M. et al. A programmable dual-RNA-guided DNA endonuclease in adaptive bacterial immunity. *Science* **337**, 816–821 (2012). 22745249 10.1126/science.1225829 PMC6286148

[2] Raguram, A., Banskota, S. & Liu, D. R. Therapeutic in vivo delivery of gene editing agents. *Cell* **185**, 2806–2827 (2022). 35798006 10.1016/j.cell.2022.03.045 PMC9454337

[3] Wang, J. Y. & Doudna, J. A. CRISPR technology: a decade of genome editing is only the beginning. *Science* **379**, eadd8643 (2023). 36656942 10.1126/science.add8643

[4] Chen, J. J. et al. CRISPR-powered optothermal nanotweezers: diverse bio-nanoparticle manipulation and single nucleotide identification. *Light Sci. Appl.* **12**, 273 (2023). 37973904 10.1038/s41377-023-01326-9 PMC10654382

[5] Zhuo, C. Y. et al. Spatiotemporal control of CRISPR/Cas9 gene editing. *Signal Transduct. Target. Ther.* **6**, 238 (2021). 34148061 10.1038/s41392-021-00645-w PMC8214627

[6] Polstein, L. R. & Gersbach, C. A. A light-inducible CRISPR-Cas9 system for control of endogenous gene activation. *Nat. Chem. Biol.* **11**, 198–200 (2015). 25664691 10.1038/nchembio.1753 PMC4412021

[7] Kim, J. H. et al. LADL: light-activated dynamic looping for endogenous gene expression control. *Nat. Methods* **16**, 633–639 (2019). 31235883 10.1038/s41592-019-0436-5 PMC6599567

[8] Nihongaki, Y. et al. CRISPR-Cas9-based photoactivatable transcription system. *Chem. Biol.* **22**, 169–174 (2015). 25619936 10.1016/j.chembiol.2014.12.011

[9] Nihongaki, Y. et al. Photoactivatable CRISPR-Cas9 for optogenetic genome editing. *Nat. Biotechnol.* **33**, 755–760 (2015). 26076431 10.1038/nbt.3245

[10] Nihongaki, Y. et al. CRISPR–Cas9-based photoactivatable transcription systems to induce neuronal differentiation. *Nat. Methods* **14**, 963–966 (2017). 28892089 10.1038/nmeth.4430

[11] Kawano, F. et al. Engineered pairs of distinct photoswitches for optogenetic control of cellular proteins. *Nat. Commun.* **6**, 6256 (2015). 25708714 10.1038/ncomms7256

[12] Hemphill, J. et al. Optical control of CRISPR/Cas9 gene editing. *J. Am. Chem. Soc.* **137**, 5642–5645 (2015). 25905628 10.1021/ja512664v PMC4919123

[13] Zhou, W. Y. & Deiters, A. Conditional control of CRISPR/Cas9 function. *Angew. Chem. Int. Ed.* **55**, 5394–5399 (2016). 10.1002/anie.201511441 26996256

[14] Lan, T. H. et al. Optogenetics for transcriptional programming and genetic engineering. *Trends Genet.* **38**, 1253–1270 (2022). 35738948 10.1016/j.tig.2022.05.014 PMC10484296

[15] Jain, P. K. et al. Development of light-activated CRISPR using guide RNAs with photocleavable protectors. *Angew. Chem. Int. Ed.* **55**, 12440–12444 (2016). 10.1002/anie.201606123 PMC5864249 27554600

[16] Liu, Y. et al. Very fast CRISPR on demand. *Science* **368**, 1265–1269 (2020). 32527834 10.1126/science.aay8204 PMC7608738

[17] Kuwasaki, Y. et al. A red light–responsive photoswitch for deep tissue optogenetics. *Nat. Biotechnol.* **40**, 1672–1679 (2022). 35697806 10.1038/s41587-022-01351-w

[18] Shao, J. W. et al. Synthetic far-red light-mediated CRISPR-dCas9 device for inducing functional neuronal differentiation. *Proc. Natl Acad. Sci. USA* **115**, E6722–E6730 (2018). 29967137 10.1073/pnas.1802448115 PMC6055150

[19] Yu, Y. H. et al. Engineering a far-red light–activated split-Cas9 system for remote-controlled genome editing of internal organs and tumors. *Sci. Adv.* **6**, eabb1777 (2020). 32923591 10.1126/sciadv.abb1777 PMC7455487

[20] Pan, Y. C. et al. Near-infrared upconversion–activated CRISPR-Cas9 system: a remote-controlled gene editing platform. *Sci. Adv.* **5**, eaav7199 (2019). 30949579 10.1126/sciadv.aav7199 PMC6447385

[21] Chi, J. D. et al. A CRISPR-Cas9-based near-infrared upconversion-activated DNA methylation editing system. *ACS Appl. Mater. Interfaces* **13**, 6043–6052 (2021). 33525876 10.1021/acsami.0c21223

[22] Chen, X. H. et al. Near-infrared optogenetic engineering of photothermal nanoCRISPR for programmable genome editing. *Proc. Natl Acad. Sci. USA* **117**, 2395–2405 (2020). 31941712 10.1073/pnas.1912220117 PMC7007568

[23] Rebelo, C. et al. Efficient spatially targeted gene editing using a near-infrared activatable protein-conjugated nanoparticle for brain applications. *Nat. Commun.* **13**, 4135 (2022). 35840564 10.1038/s41467-022-31791-6 PMC9287341

[24] Zhou, Y. et al. A small and highly sensitive red/far-red optogenetic switch for applications in mammals. *Nat. Biotechnol.* **40**, 262–272 (2022). 34608325 10.1038/s41587-021-01036-w

[25] Gorka, A. P. et al. A near-IR uncaging strategy based on cyanine photochemistry. *J. Am. Chem. Soc.* **136**, 14153–14159 (2014). 25211609 10.1021/ja5065203 PMC4195383

[26] Pollock, R. & Clackson, T. Dimerizer-regulated gene expression. *Curr. Opin. Biotechnol.* **13**, 459–467 (2002). 12459338 10.1016/s0958-1669(02)00373-7

[27] Serkova, N. et al. Sirolimus, but not the structurally related RAD (everolimus), enhances the negative effects of cyclosporine on mitochondrial metabolism in the rat brain. *Br. J. Pharmacol.* **133**, 875–885 (2001). 11454661 10.1038/sj.bjp.0704142 PMC1572850

[28] Ho, S. N. et al. Dimeric ligands define a role for transcriptional activation domains in reinitiation. *Nature* **382**, 822–826 (1996). 8752278 10.1038/382822a0

[29] Banaszynski, L. A., Liu, C. W. & Wandless, T. J. Characterization of the FKBP·rapamycin·FRB ternary complex. *J. Am. Chem. Soc.* **127**, 4715–4721 (2005). 10.1021/ja043277y 15796538

[30] Zetsche, B., Volz, S. E. & Zhang, F. A split-Cas9 architecture for inducible genome editing and transcription modulation. *Nat. Biotechnol.* **33**, 139–142 (2015). 25643054 10.1038/nbt.3149 PMC4503468

[31] Brown, K. A. et al. Light-cleavable rapamycin dimer as an optical trigger for protein dimerization. *Chem. Commun.* **51**, 5702–5705 (2015). 10.1039/c4cc09442e 25716548

[32] Choi, J. et al. Structure of the FKBP12-rapamycin complex interacting with binding domain of human FRAP. *Science* **273**, 239–242 (1996). 8662507 10.1126/science.273.5272.239

[33] Karginov, A. V. et al. Light regulation of protein dimerization and kinase activity in living cells using photocaged rapamycin and engineered FKBP. *J. Am. Chem. Soc.* **133**, 420–423 (2011). 21162531 10.1021/ja109630v PMC3133816

[34] Wagner, R. et al. Rapamycin analogs with reduced systemic exposure. *Bioorg. Med. Chem. Lett.* **15**, 5340–5343 (2005). 16185865 10.1016/j.bmcl.2005.06.106

[35] Nani, R. R. et al. Near-IR light-mediated cleavage of antibody-drug conjugates using cyanine photocages. *Angew. Chem. Int. Ed.* **54**, 13635–13638 (2015). 10.1002/anie.201507391 PMC4743669 26403799

[36] Dixon, A. S. et al. NanoLuc complementation reporter optimized for accurate measurement of protein interactions in cells. *ACS Chem. Biol.* **11**, 400–408 (2016). 26569370 10.1021/acschembio.5b00753

[37] Perez-Pinera, P. et al. RNA-guided gene activation by CRISPR-Cas9-based transcription factors. *Nat. Methods* **10**, 973–976 (2013). 23892895 10.1038/nmeth.2600 PMC3911785

[38] Fegan, A. et al. Chemically controlled protein assembly: techniques and applications. *Chem. Rev.* **110**, 3315–3336 (2010). 20353181 10.1021/cr8002888

[39] Dow, L. E. et al. Inducible in vivo genome editing with CRISPR-Cas9. *Nat. Biotechnol.* **33**, 390–394 (2015). 25690852 10.1038/nbt.3155 PMC4390466

[40] Zuris, J. A. et al. Cationic lipid-mediated delivery of proteins enables efficient protein-based genome editing in vitro and in vivo. *Nat. Biotechnol.* **33**, 73–80 (2015). 25357182 10.1038/nbt.3081 PMC4289409

[41] Johansson, A. C. et al. Cathepsin D mediates cytochrome *c* release and caspase activation in human fibroblast apoptosis induced by staurosporine. *Cell Death Differ.* **10**, 1253–1259 (2003). 14576777 10.1038/sj.cdd.4401290

[42] Chen, J. C. et al. Saikosaponin-a induces apoptotic mechanism in human breast MDA-MB-231 and MCF-7 cancer cells. *Am. J. Chin. Med.* **31**, 363–377 (2003). 12943168 10.1142/S0192415X03001065

[43] Moroz-Omori, E. V. et al. Photoswitchable gRNAs for spatiotemporally controlled CRISPR-Cas-based genomic regulation. *ACS Cent. Sci.* **6**, 695–703 (2020). 32490186 10.1021/acscentsci.9b01093 PMC7256956

[44] Aksoy, Y. A. et al. Spatial and temporal control of CRISPR-Cas9-mediated gene editing delivered via a light-triggered liposome system. *ACS Appl. Mater. Interfaces* **12**, 52433–52444 (2020). 33174413 10.1021/acsami.0c16380

[45] Piazena, H., Meffert, H. & Uebelhack, R. Spectral remittance and transmittance of visible and infrared-a radiation in human skin—comparison between in vivo measurements and model calculations. *Photochem. Photobiol.* **93**, 1449–1461 (2017). 28471473 10.1111/php.12785

[46] Yazdanfar, S. et al. Multiphoton microscopy with near infrared contrast agents. *J. Biomed. Opt.* **15**, 030505 (2010). 20614991 10.1117/1.3420209 PMC2881927

[47] Berezin, M. Y. et al. Two-photon optical properties of near-infrared dyes at 1.55 μm excitation. *J. Phys. Chem. B* **115**, 11530–11535 (2011). 21866928 10.1021/jp207618e PMC3233988

[48] Hsu, P. D. et al. DNA targeting specificity of RNA-guided Cas9 nucleases. *Nat. Biotechnol.* **31**, 827–832 (2013). 23873081 10.1038/nbt.2647 PMC3969858

[49] Kaida, D. et al. U1 snRNP protects pre-mRNAs from premature cleavage and polyadenylation. *Nature* **468**, 664–668 (2010). 20881964 10.1038/nature09479 PMC2996489

[50] Ran, F. A. et al. Genome engineering using the CRISPR-Cas9 system. *Nat. Protoc.* **8**, 2281–2308 (2013). 24157548 10.1038/nprot.2013.143 PMC3969860

[51] Schmittgen, T. D. & Livak, K. J. Analyzing real-time PCR data by the comparative *C*_T_ method. *Nat. Protoc.* **3**, 1101–1108 (2008). 18546601 10.1038/nprot.2008.73

[52] Chalfoun, J. et al. MIST: accurate and scalable microscopy image stitching tool with stage modeling and error minimization. *Sci. Rep.* **7**, 4988 (2017). 28694478 10.1038/s41598-017-04567-y PMC5504007

