# Supplementary material for: Near-infrared light activatable chemically induced CRISPR system
Source: Light Sci Appl. 2025 Jul 1;14:229. doi: 10.1038/s41377-025-01917-8 (PMC12214668; doi:10.1038/s41377-025-01917-8)
Supplement: Supplementary file 1 — Supplementary Information for Near-infrared light activatable chemically induced CRISPR system [file 41377_2025_1917_MOESM1_ESM.pdf]

Supplementary Information for

Near-infrared light activatable chemically induced CRISPR system

Lei Zhang[1†*], Xuejun Zhang[1†], Le Qiu[1†], Song Mao[2], Jia Sheng[2*], Liming Chen[1], Umar Khan[1], Paul K. Upputuri[1], Yuri N. Zakharov[1], Mark F. Coughlan[1] and Lev T. Perelman[1*]

[1]*Center for Advanced Biomedical Imaging and Photonics, Division of Gastroenterology, Department of Medicine, Beth Israel Deaconess Medical Center, Harvard University, Boston 02215, USA*

[2]*The RNA Institute, Department of Chemistry, University at Albany, Albany 12222, USA*

*Corresponding Authors, Emails: lzhang11@bidmc.harvard.edu; jsheng@albany.edu; ltperel@bidmc.harvard.edu

[†]These authors contributed equally to this work

Supplementary Note 1. Synthesis procedure

Anhydrous solvents were used and redistilled following standard procedures. All solid reagents were dried under high vacuum prior to use. Air-sensitive reactions were conducted under an argon atmosphere. Reaction progress was monitored using analytical thin-layer chromatography (TLC) plates pre-coated with silica gel F254 (Dynamic Adsorbents) and visualized under UV light. Flash column chromatography was performed using silica gel (32–63 μm). All ^{1}H, ^{13}C and ^{31}P NMR spectra were recorded on a Bruker 500 MHz spectrometer, with chemical shift values reported in ppm. ^{13}C NMR signals were identified using the APT technique. High-resolution mass spectrometry (HRMS) was performed using QTOF-MS at the University at Albany, SUNY.

Procedure for synthesis of S5[1,2], S7[1], S10[3] and S12[4]

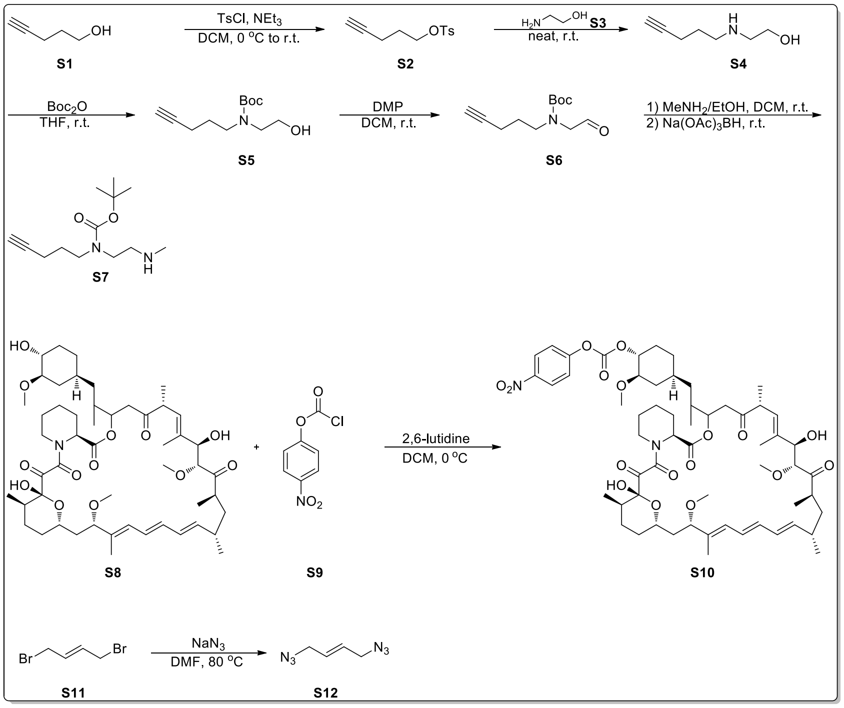

Scheme S1. Synthesis of *S7, S10 and S12*

***pent-4-yn-1-yl 4-methylbenzenesulfonate* (S2):** A solution of **S1** (5.04 g, 60 mmol) and TsCl (13.5 g, 72 mmol) in DCM (200 mL) was cooled to 0 °C. Triethylamine (Et_3N, 15 mL, 90 mmol) was added dropwise, and the mixture was stirred overnight at room temperature. The reaction mixture was then diluted with DCM (300 mL), washed sequentially with water (200 mL) and brine (200 mL), dried over anhydrous Na_2SO_4, filtered, and concentrated under reduced pressure. The resulting residue was purified by silica gel chromatography (hexane/ethyl acetate, 5:1) to yield S2 (13.5 g, 56 mmol, 93%) as a yellow oil.

***2-(pent-4-yn-1-ylamino)ethanol* S4**: Ethanolamine **S3** (20 mL, 336 mmol) was added gradually to S2 (13.5 g, 56 mmol) with stirring. The mixture was stirred neat at room temperature for 3 hours. The reaction was then diluted with water (150 mL) and brine (150 mL) and extracted with ethyl acetate (10 × 100 mL). The combined organic layers were dried over anhydrous Na_2SO_4, filtered, and concentrated under reduced pressure to yield crude S4 (6.2 g, 49 mmol), which was used in the next step without further purification.

***tert-butyl (2-hydroxyethyl)(pent-4-yn-1-yl)carbamate* S5**: To a solution of crude **S4** (6.2 g, 49 mmol) in THF (15 mL), triethylamine (Et_3N, 14 mL, 98 mmol) and di-tert-butyl dicarbonate (Boc_2O, 11.7 g, 54 mmol) were added. The reaction mixture was stirred at room temperature for 3 hours. After removing the solvent under reduced pressure, the residue was purified by silica gel chromatography (dichloromethane/methanol, 10:1) to yield S5 (4.7 g, 20.7 mmol, 42%) as a yellow oil. ^{1}H NMR (500 MHz, $CDCl_3$) δ 3.75 (t, J = 5.5 Hz, 2H), 3.40 (t, J = 5.0 Hz, 2H), 3.33 (t. J = 7.5 Hz, 2H), 2.21 (td, J = 3.0, 7.0 Hz, 2H), 1.96 (t, J = 2.5 Hz, 1H), 1.79-1.73 (m, 2H), 1.46 (s, 9H).

***tert-butyl (2-oxoethyl)(pent-4-yn-1-yl)carbamate* S6**: To a solution of **S5** (1.6 g, 7 mmol) in DCM (36 mL), Dess-Martin periodinane (3.2 g, 7.35 mmol) was added. The mixture was stirred at room temperature for 1 hour. The reaction was then diluted with ethyl acetate (200 mL) and washed sequentially with saturated Na_2SO_3 solution (2 × 50 mL), saturated $NaHCO_3$ solution (200 mL), and brine (200 mL). The organic layer was dried over anhydrous Na_2SO_4, filtered, and concentrated under reduced pressure. The residue was purified by silica gel chromatography (hexane/ethyl acetate, 10:1) to yield S6 (930 mg, 4.1 mmol, 59%) as a yellow oil. ^{1}H NMR (500 MHz, $CDCl_3$) δ 9.55 (s, 1H), 3.96-3.86 (m, 2H), 3.39-3.35 (m, 2H), 2.21 (m, 2H), 1.95-1.94 (m, 1H), 1.72 (m, 2H), 1.46-1.39 (m, 9H).

***tert-butyl (2-(methylamino)ethyl)(pent-4-yn-1-yl)x* S7**. To a solution of **S6** (930 mg, 4.1 mmol) in DCM (50 mL), methylamine (2.1 mL, 16.4 mmol, 33 wt% in ethanol) was added. The mixture was stirred at room temperature for 30 minutes, after which sodium triacetoxyborohydride ($Na(OAc)_3BH$, 1.4 g, 6.2 mmol) was added. The reaction mixture was stirred for an additional 2 hours. The reaction was then diluted with DCM (100 mL) and washed with 1 N NaOH (150 mL). The organic layer was dried over anhydrous Na_2SO_4, filtered, and concentrated under reduced pressure. The residue was purified by silica gel chromatography (dichloromethane/methanol, 4:1) to yield **S7** (700 mg, 2.9 mmol, 71%) as a yellow oil. ^{1}H NMR (500 MHz, $CDCl_3$) δ 3.36 (m, 2H), 3.28 (t, J = 7.5 Hz, 2H), 2.82 (m, 2H), 2.48 (s, 3H), 2.17 (td, J = 2.5, 7.0 Hz, 2H), 1.95-1.93 (m, 1H), 1.74-1.71 (m, 2H), 1.43 (s, 9H).

***PNP_Activated Rapamycin* S10**: To a solution of **Rapamycin** (180 mg, 0.2 mmol) in DCM (2 mL), 4-nitrophenyl chloroformate (80 mg, 0.4 mmol) was added

at 0 °C. Subsequently, 2,6-lutidine (0.1 mL, 0.8 mmol) was added dropwise. After stirring for 30 minutes at 0 °C, the reaction was diluted with DCM (50 mL) and washed sequentially with saturated $NaHCO_3$ solution (50 mL) and brine (50 mL). The organic layer was dried over anhydrous Na_2SO_4, filtered, and concentrated under reduced pressure. The residue was purified by silica gel chromatography (hexane/ethyl acetate, 1:1) to yield **S10** (165 mg, 0.15 mmol, 75%) as a white solid. ^1H NMR (500 MHz, $CDCl_3$) δ 8.27 (d, J = 9.0 Hz, 2H), 7.39 (d, J = 9.0 Hz, 2H), 6.41-6.11 (m, 3H), 5.98-5.87 (m, 1H), 5.56-5.40 (m, 2H), 5.28 (m, 1H), 5.19-5.10 (m, 1H), 4.76-4.55 (m, 2H), 4.23-4.09 (m, 1H), 3.90-3.85 (m, 1H), 3.74-3.55 (m, 3H), 3.44 (s, 3H), 3.34 (s, 3H), 3.28-3.22 (m, 1H), 3.13 (s, 3H), 2.89-2.55 (m, 3H), 2.35-2.15 (m, 4H), 2.08-1.96 (m, 2H), 1.89-1.68 (m, 9H), 1.66-1.39 (m, 15H), 1.37-1.17 (m, 9H), 1.17-0.81 (m, 27H). HRMS (ESI-TOF): m/z calculated for $C_{58}H_{82}N_2NaO_{17}$ $(M+Na)^+$: 1101.5511, observed: 1101.5491.

(E)-1,4-diazidobut-2-ene **S12**: A mixture of (E)-1,4-dibromobut-2-ene **S11** (1 g, 5 mmol) and NaN_3 (3.25 g, 50 mmol) in DMF (20 mL) was stirred at 80 °C overnight. The reaction mixture was cooled to room temperature, diluted with water (100 mL), and extracted with hexanes (3 × 100 mL). The combined organic layers were washed with brine (200 mL), dried over anhydrous Na_2SO_4, filtered, and concentrated under reduced pressure. The residue was purified by silica gel chromatography (hexane/ethyl acetate, 20:1) to yield **S12** (300 mg, 2.2 mmol, 43%) as a colorless oil. ^1H NMR (500 MHz, $CDCl_3$) δ 5.83-5.81 (m, 2H), 3.84-3.83 (m, 4H).

Compound **2**: A mixture of **1** (**IR780**, 333 mg, 0.5 mmol), **S7** (360 mg, 1.5 mmol) and DIPEA (0.4 mL, 2.5 mmol) was stirred for 1 h at 105 °C. The green solution

was changed to dark blue. After cooling to room temperature, 4-(trifluoromethyl)-benzyl (0.25 mL, 0.5 mmol) and another DIPEA (0.4 mL, 2.5 mmol) were added. The mixture was stirred for another 3 h and diluted with DCM (100 mL), washed with saturated $NaHCO_3$ (100 mL) and brine (100 ml). The organic layer was dried over anhydrous Na_2SO_4, filtered and subsequently concentrated under reduced pressure. The residue was then purified by silica gel chromatography (dichloromethane/methanol = 20:1) to afford **2** (340 mg, 0.39 mmol, 78% yield) as a dark blue solid. ^{1}H NMR (500 MHz, CD_3OD) δ 7.25 (d, *J* = 13.5 Hz, 2H), 7.45 (d, *J* = 7.5 Hz, 2H), 7.20-7.18 (m, 4H).6.02 (m, 2H), 4.03 (m, 4H), 3.91 (m, 2H), 3.49 (s, 3H), 3.01 (m, 2H), 2.88 (m, 2H), 2.56 (m, 4H), 2.3 (m, 1H), 2.23-2.19 (m, 6H), 1.77-1.73 (m, 4H), 1.71 (s, 12 H), 1.43 (s, 9H), 1.06 (t, *J* = 7.5 Hz, 6H). HRMS (ESI-TOF): m/z calculated for $C_{49}H_{67}N_4O_2$ $(M-I)^+$: 743.5259, observed: 743.5282.

Compound **4**: A mixture of compound **2** (170 mg, 0.2 mmol) and neat trifluoroacetic acid (TFA, 2 mL) was stirred for 1 h at room temperature. The red solution was concentrated and co-evaporated with DCM (10 mL) for several times until the residue was changed back to dark blue. The intermediate dark blue compound **3** was then dissolved in pyridine (5 mL), **S10** (430 mg, 0.4 mmol) and DMAP (49 mg, .0.4 mmol) were added. The mixture was stirred overnight at room temperature. After concentration, the residue was purified by silica gel chromatography (dichloromethane/methanol = 20:1) to afford **4** (72 mg, 0.04 mmol, 20% yield) as a blue solid. ^{1}H NMR (500 MHz, CD_3OD) δ 7.79 (d, *J* = 13.5 Hz, 2 H), 7.48 (d, *J* = 7.5 Hz, 2 H), 7.41-7.37 (m, 2H), 7.23-7.21 (m, 4H), 6.49-6.43 (m, 1H), 6.32-6.00 (m, 6H), 5.51-5.45 (m, 1H), 5.26-5.23 (m, 2H), 5.08 (m, 1H),

4.47 (m, 2H), 4.18-4.06 (m, 7H), 3.83 (m, 4H), 3.71-3.46 (5H), 3.29-3.13 (m, 8H), 2.96 (m, 3H), 2.85-2.80 (m, 1H), 2.58 (m, 5H), 2.50-2.44 (m, 1H), 2.33-2.29 (m, 2H), 2.18-1.98 (m, 7H), 1.92-1.84 (m, 10 H), 1.72 (m, 12 H), 1.62 (m, 2H), 1.48-1.24 (m, 9H), 1.19-0.85 (m, 26H). HRMS (ESI-TOF): m/z calculated for $C_{96}H_{136}N_5O_{14}$ $(M-I)^+$: 1583.0078, observed: 1583.0069.

Compound **5**: A mixture of **4** (30 mg, 0.017 mmol), **S12** (1.2 mg, 0.0085 mmol), $CuSO_4{\cdot}5H_2O$ (1 mg, 0.004 mmol) and sodium ascorbate (1.6 mg, 0.008 mmol) in DCM/*t*-BuOH/H_2O (1:1:1, 3 mL) was stirred overnight at 40 °C. The reaction was diluted with DCM (50 mL), washed with brine (50 mL), dried over anhydrous Na_2SO_4, filtered and subsequently concentrated under reduced pressure. The residue was then purified by silica gel chromatography (dichloromethane/methanol = 8:1) to afford **5** (10 mg, 0.0028 mmol, 33% yield) as a blue solid. ^1H NMR (500 MHz, CD_3OD) δ 7.77-7.74 (m, 4H), 7.50-7.44 (m, 4H), 7.39-7.35 (m, 4H), 7.21-7.17 (m, 8H), 6.49-6.43 (m, 2H), 6.32-5.90 (m, 10H), 5.51-5.45 (m, 2H), 5.25-5.22 (m, 2H), 5.06 (m, 2H), 4.97 (m, 2H), 4.47 (m, 3H), 4.19-4.04 (m, 11H), 3.82-3.55 (m, 15H), 3.30-3.13 (m, 17H), 2.94 (m, 6H), 2.84-2.43 (m, 10H), 2.29-1.84 (m, 32 H), 1.74-1.63 (m, 36H), 1.50-0.84 (m, 74H). HRMS (ESI-TOF): m/z calculated for $C_{196}H_{278}N_{16}O_{28}/2$ $(M-2I)^{2+}/2$: 1652.0405, observed: 1652.0446.

^{1}HNMR, ^{13}CNMR and Mass Spectrum

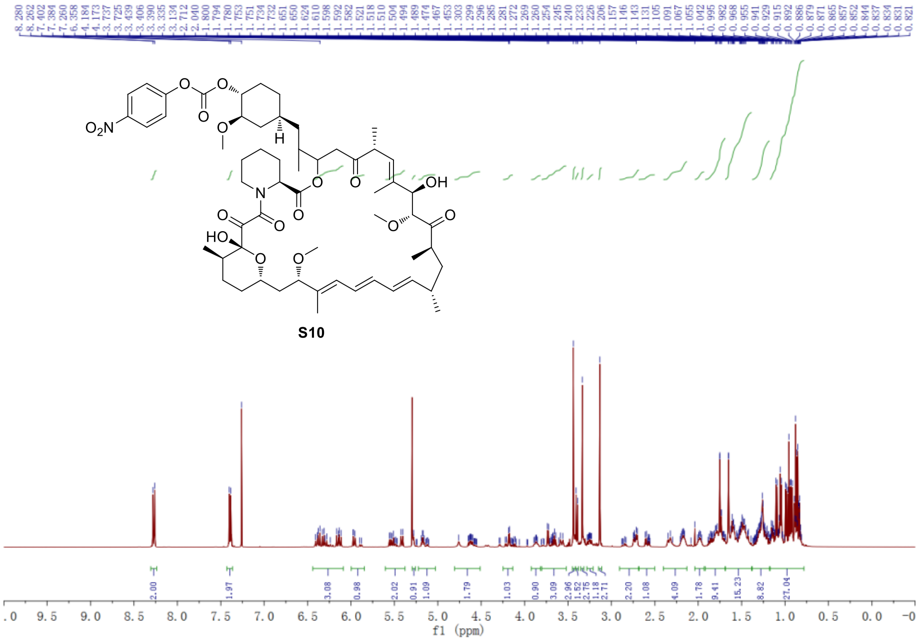

Fig. S1. ^{1}H NMR of PNP-activated rapamycin (compound **S10**).

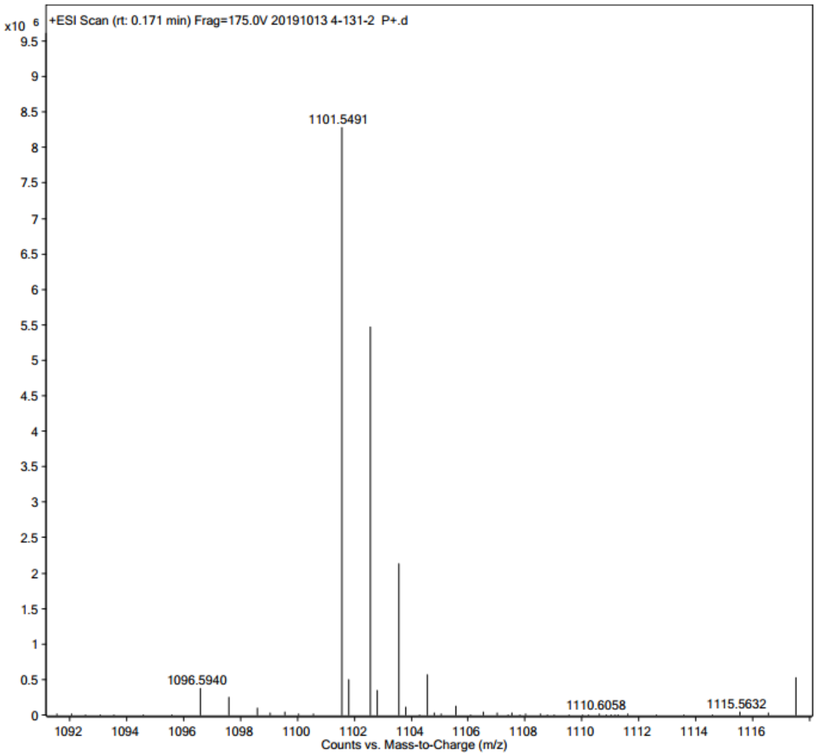

Fig. S2. Mass of PNP-activated rapamycin (compound **S10**).

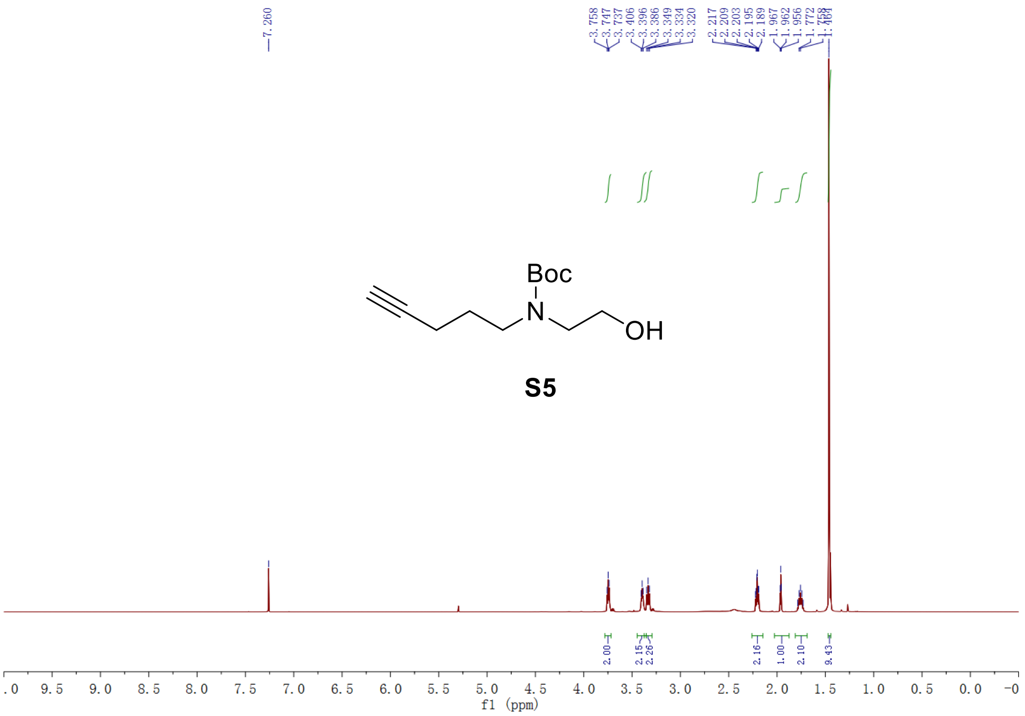

Fig. S3. ^{1}H NMR of compound **S5**.

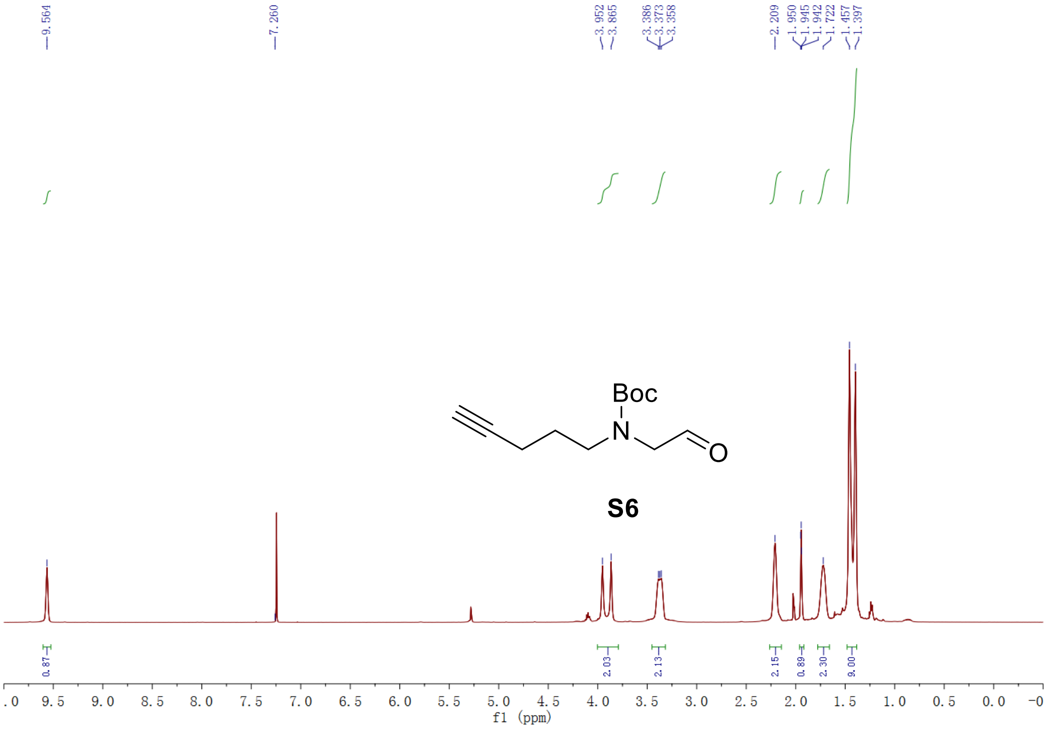

Fig. S4. ^{1}H NMR of compound **S6**.

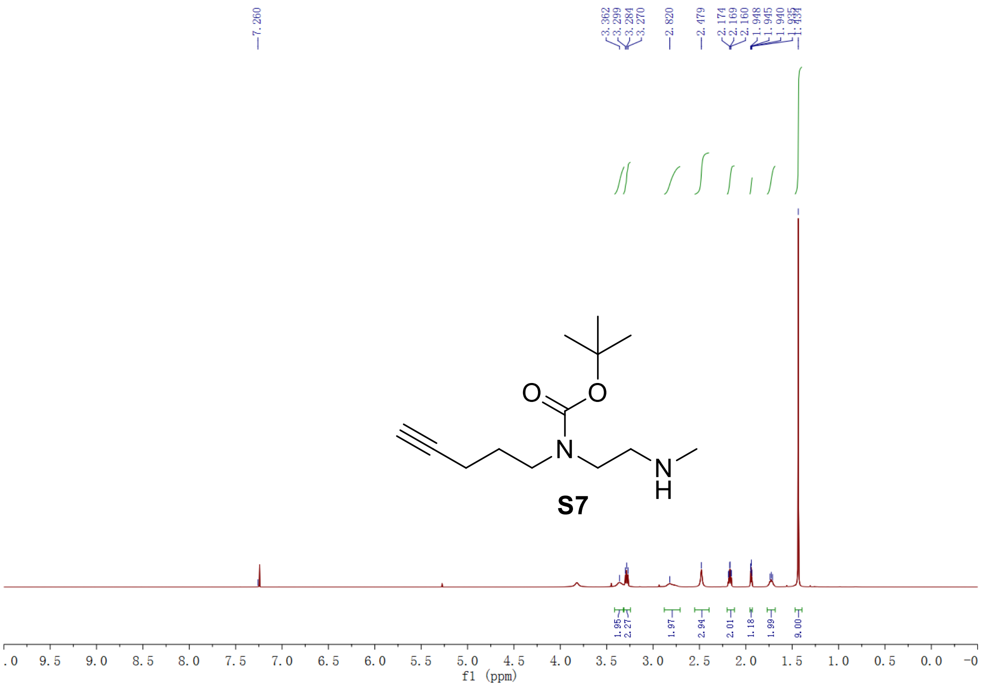

Fig. S5. ^{1}H NMR of compound **S7**.

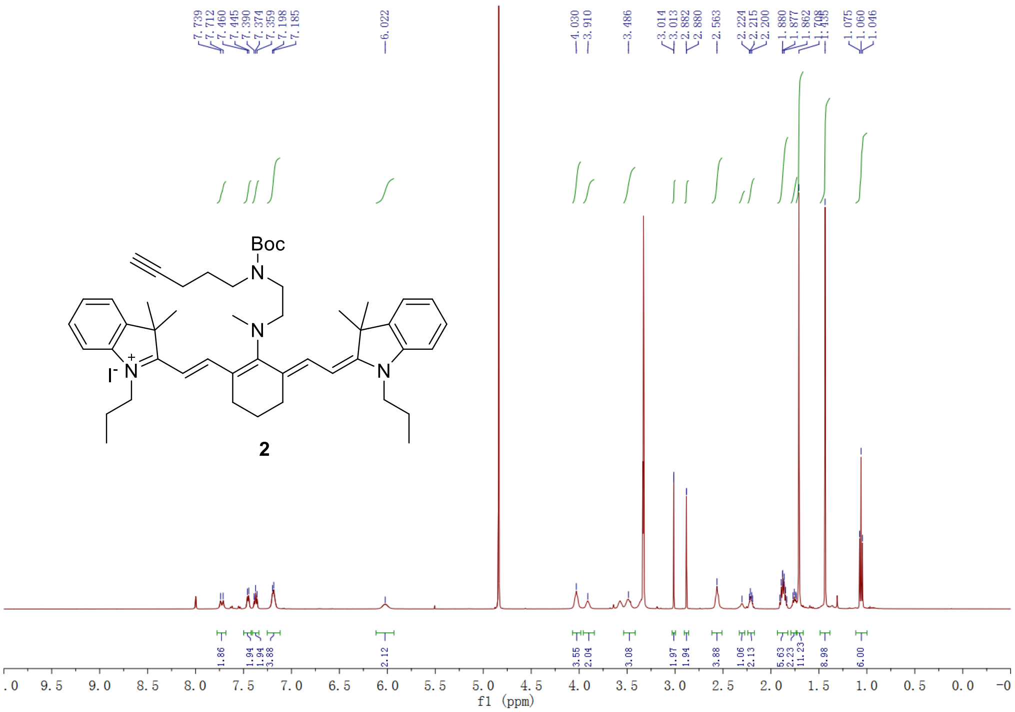

Fig. S6. ^{1}H NMR of C4′−N linked IR780-carbamate (compound **2**).

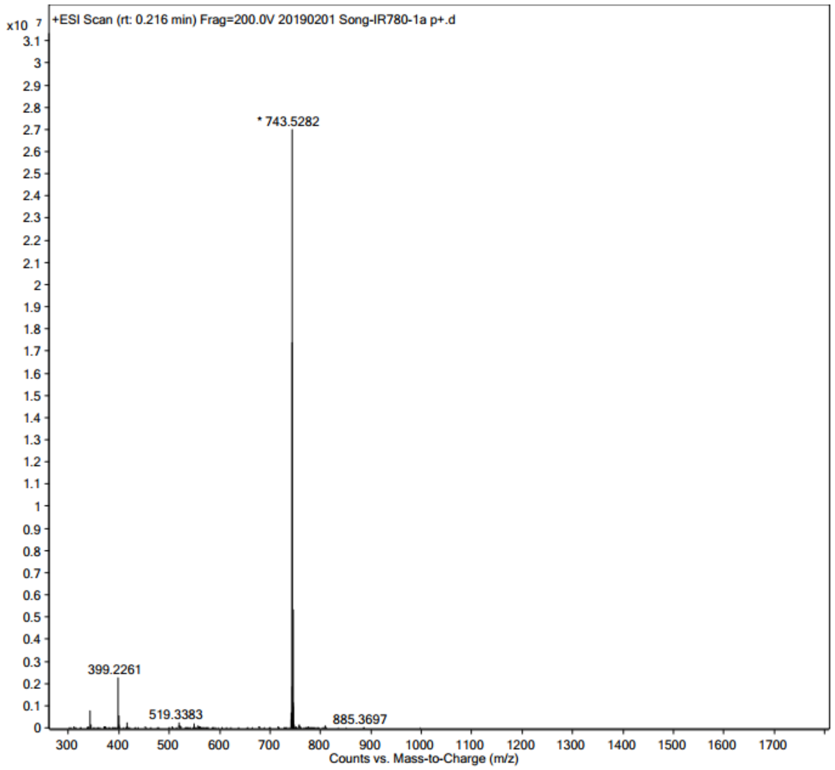

Fig. S7. Mass of C4′−N linked IR780-carbamate (compound **2**).

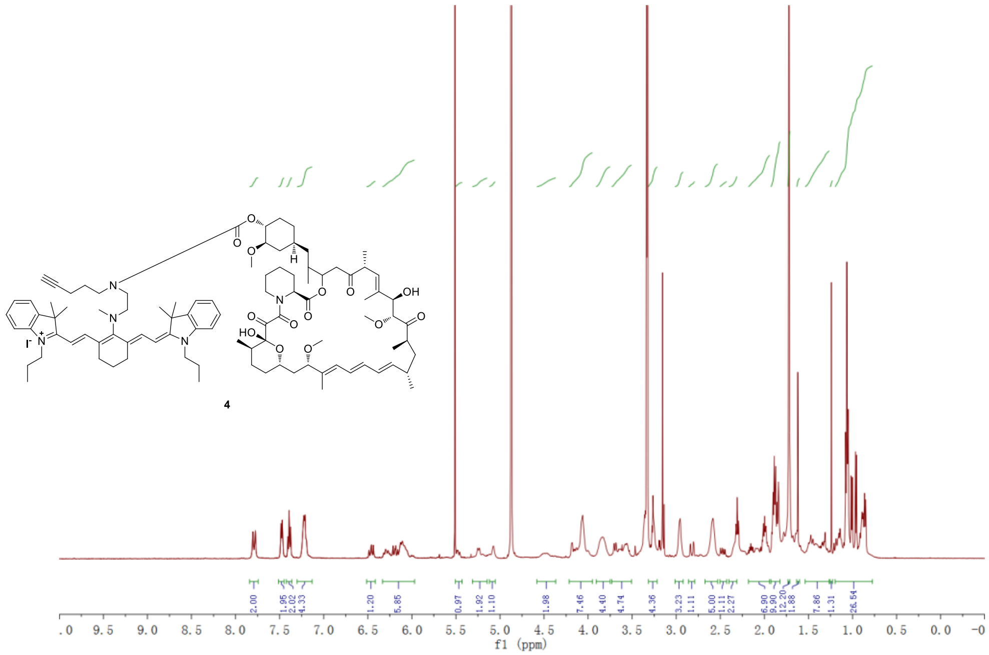

Fig. S8. ^{1}H NMR of compound **4**.

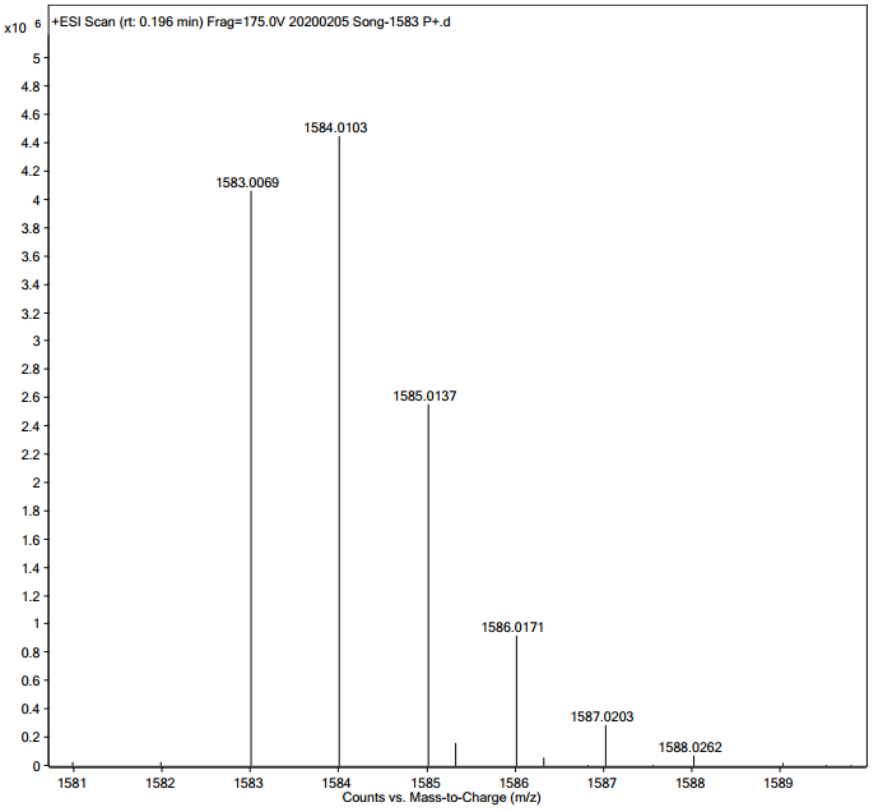

Fig. S9. Mass of compound **4**.

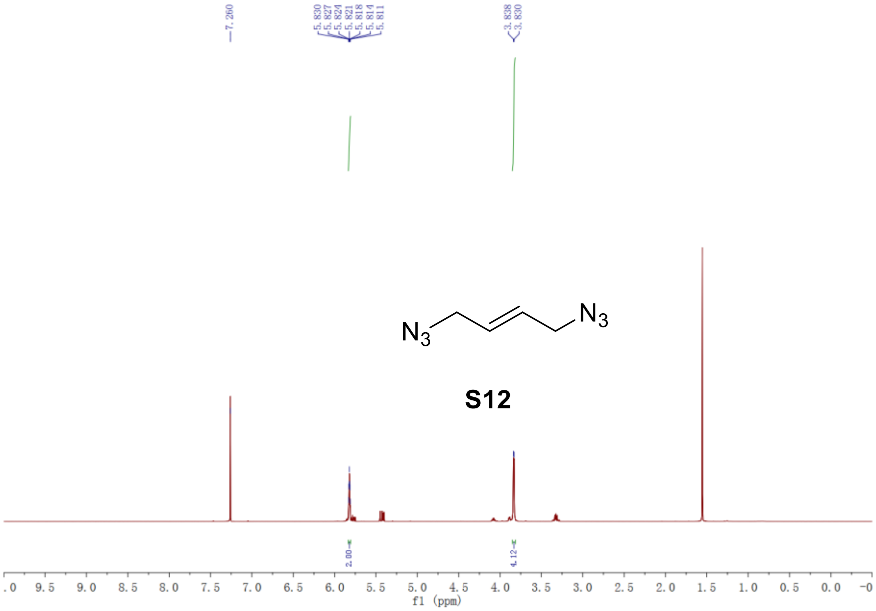

Fig. S10. ^{1}H NMR of compound **S12**.

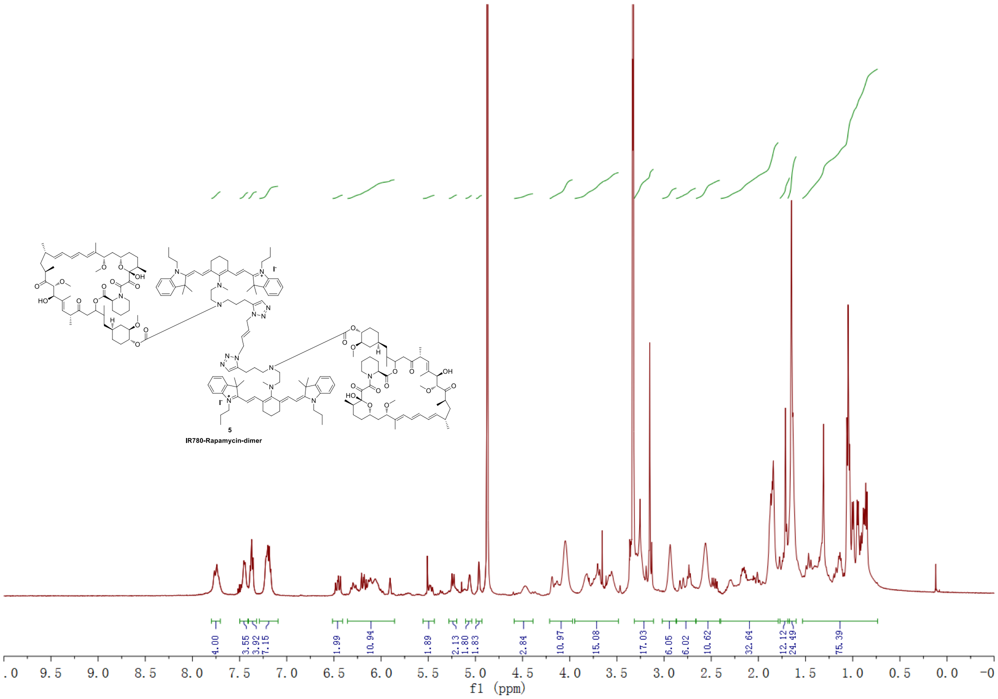

Fig. S11. ^{1}H NMR of IR780-bridged dimers (compound **5**).

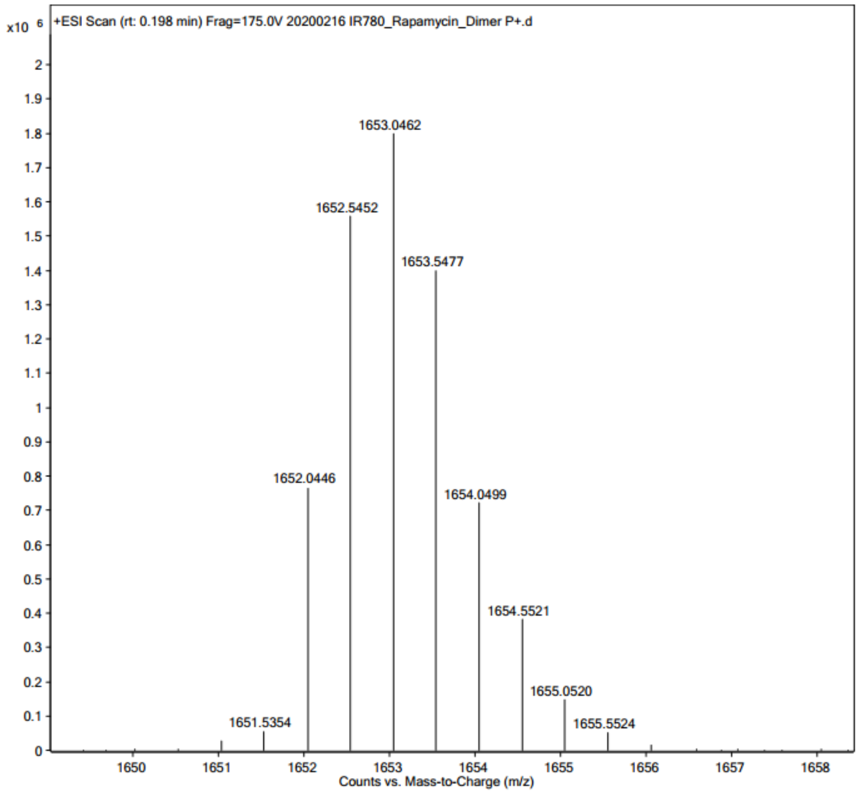

Fig. S12. Mass of IR780-bridged dimers (compound **5**).

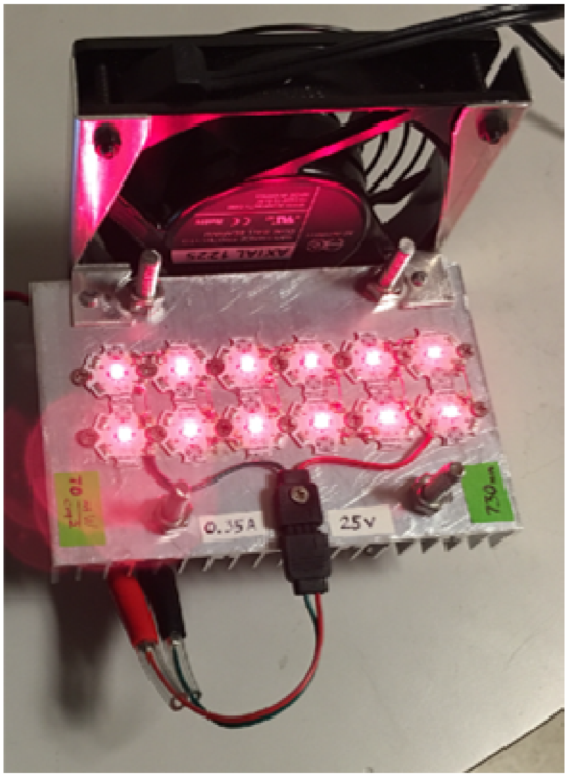

Fig. S13. LED array for photoactivation. The LED array consisted of six 730 nm LEDs (LST1-01G01-FRD1-00, Opulent Americas) mounted on a 130 mm × 90 mm aluminum board (McMaster). The LEDs were wired in two series, with each series connected in parallel on the same board. The array was powered by a DC Power Supply (TP3016M, Tekpower). During illumination, a 24-well plate was positioned 1 cm above the LED array, allowing the upward-directed light to uniformly illuminate the bottoms of the central 12 wells of the plate.

Supplementary Note 2. Digitonin-based reversible permeabilization method

To enhance the ability of the IR780-bridged rapamycin dimer to cross the cell membrane, we developed a safe and reversible permeabilization method using digitonin. Digitonin is a mild detergent that interacts specifically with membrane cholesterol, temporarily increasing the pore size of the cell membrane, thereby allowing larger molecules to pass through without causing long-term damage to the cells.

In this method, transfected cells were treated with 10 μg mL^{-1} digitonin (Thermo Fisher Scientific), which was dissolved in a hypotonic solution (Rainbow Scientific), for 15 minutes (37 °C, 5% CO_2). The hypotonic solution aids in creating an osmotic gradient that enhances the permeabilization effect of digitonin. Following this brief incubation, the digitonin solution was carefully removed to avoid prolonged exposure that could compromise cell viability. Subsequently, the IR780-bridged rapamycin dimer (100 nM) or rapamycin alone (used as a positive control) was added to the cells in fresh medium. The cells were incubated for 20 minutes under standard cell culture conditions (37 °C, 5% CO_2) to allow sufficient uptake of the compounds. After this incubation, cell culture medium containing 4 mM $CaCl_2$ was introduced to the cells and further incubated for 20 min to facilitate any downstream processes and to stabilize cellular conditions before illumination.

This method provides a reliable and controlled approach for delivering large or complex molecules such as the IR780-bridged rapamycin dimer into cells, ensuring efficient internalization while maintaining cell integrity and function.

Table S1. Guides used for directing cas9/dCas9 binding.

**Primer name**	**Guide sequence (5˙-3˙)**	**Reference**
OPTN sg	GGAGATGCAGAGTCGTCATG	This study
ASCL1 sg1	GCAGCCGCTCGCTGCAGCAG	5,6
ASCL1 sg2	ATGGAGAGTTTGCAAGGAGC	5,6
ASCL1 sg3	GGCTGGGTGTCCCATTGAAA	5,6
ASCL1 sg4	TGTTTATTCAGCCGGGAGTC	5,6
IL1RN sg1	TTGTACTCTCTGAGGTGCTC	5,6

IL1RN sg2	TACGCAGATAAGAACCAGTT	5,6
IL1RN sg3	GCATCAAGTCAGCCATCAGC	5,6
IL1RN sg4	TGAGTCACCCTCCTGGAAAC	5,6
HBG sg1	GCTAGGGATGAAGAATAAA	6
HBG sg2	TTGACCAATAGCCTTGACA	6
HBG sg3	TGCAAATATCTGTCTGAAA	6
HBG sg4	AAATTAGCAGTATCCTCTT	6
MyoD sg1	CCTGGGCTCCGGGGCGTTT	6
MyoD sg2	GGCCCCTGCGGCCACCCCG	6
MyoD sg3	CTCCCTCCCTGCCCGGTAG	6
MyoD sg4	AGGTTTGGAAAGGGCGTGC	6

References

1. Nani, R. R., *et al*. Near-IR light-mediated cleavage of antibody-drug conjugates using cyanine photocages. *Angew. Chem. Int. Ed.* **54**, 13635–13628 (2015).
2. Brown, K. A., *et al*. Light-cleavable rapamycin dimer as an optical trigger for protein dimerization. *Chem. Commun.* **51**, 5702–5705 (2015).
3. Wagner, R. *et al*. Rapamycin analogs with reduced systemic exposure. *Bioorg. Med. Chem. Lett.* **15**, 5340–5343 (2005).
4. Holstein, J. M., Schulz, D. & Rentmeister, A. Bioorthogonal site-specific labeling of the 5′-cap structure in eukaryotic mRNAs. *Chem. Commun.* **50**, 4478–4481 (2014).
5. Zetsche, B., Volz, S. E. & Zhang, F. A split-Cas9 architecture for inducible genome editing and transcription modulation. *Nat. Biotechnol.* **33**, 139–142 (2015).
6. Perez-Pinera, P. *et al.* RNA-guided gene activation by CRISPR-Cas9-based transcription factors. *Nat. Methods* **10**, 973–976 (2013).
